# Biosynthesis of Pellucidin A in *Peperomia pellucida* (L.) HBK

**DOI:** 10.3389/fpls.2021.641717

**Published:** 2021-03-22

**Authors:** Marcilio M. de Moraes, Massuo J. Kato

**Affiliations:** Institute of Chemistry, University of São Paulo, São Paulo, Brazil

**Keywords:** Piperaceae, *Peperomia pellucida*, pellucidin A, biosynthesis, [2+2] cycloaddition

## Abstract

*Peperomia pellucida* (L.) HBK (Piperaceae) (“jabuti herb”) is an herbaceous plant that is widespread in the tropics and has several ethnomedicinal uses. The phytochemical study of leaf extracts resulted in the isolation of 2,4,5-trimethoxycinnamic acid, 5,6,7-trimethoxyflavone, 2,4,5-trimethoxystyrene, 2,4,5-trimethoxybenzaldehyde, dillapiol, and sesamin in addition to pellucidin A. The co-occurrence of styrene and cyclobutane dimers suggested the formation of pellucidin A by a photochemical [2+2] cycloaddition of two molecules of 2,4,5-trimethoxystyrene. To investigate this biogenesis, analysis of plant leaves throughout ontogeny and treatments such as drought, herbivory and, exposure to jasmonic acid and UV_365_ light were carried out. Significant increases in the content of dillapiol (up to 86.0%) were found when *P. pellucida* plants were treated with jasmonic acid, whereas treatment under UV_365_ light increase the pellucidin A content (193.2%). The biosynthetic hypothesis was examined by feeding various ^13^C-labeled precursors, followed by analysis with GC-MS, which showed incorporation of L-(2-^13^C)-phenylalanine (0.72%), (8-^13^C)-cinnamic acid (1.32%), (8-^13^C)-ferulic acid (0.51%), (8-^13^C)-2,4,5-trimethoxycinnamic acid (7.5%), and (8-^13^C)-2,4,5-trimethoxystyrene (12.8%) into pellucidin A. The enzymatic conversion assays indicated decarboxylation of 2,4,5-trimethoxycinnamic acid into 2,4,5-trimethoxystyrene, which was subsequently dimerized into pellucidin A under UV light. Taken together, the biosynthesis of pellucidin A in *P. pellucida* involves a sequence of reactions starting with L-phenylalanine, cinnamic acid, ferulic acid, 2,4,5-trimethoxycinnamic acid, which then decarboxylates to form 2,4,5-trimethoxystyrene and then is photochemically dimerized to produce pellucidin A.

## Introduction

*Peperomia* is one of the most diverse genera in the Piperaceae family (Samain et al., 2009), which together with *Piper* are represented by approximately 1600 and 2000 species, respectively (Wanke et al., 2007; Samain et al., 2009). *Peperomia* species are found mainly in regions of humid or mountainous forests in Asia, Africa, Oceania, and Central and South America (Wanke et al., 2006). The plants have wide adaptability to different climatic conditions, soil types, and highland environments (Smith et al., 2008), and are also extensively cultivated for ornamental purposes.

Most *Peperomia* species present leaves with specialized tissues for water storage, with the degree of succulence varying considerably according to foliar morphology (Kaul, 1997) and several geophytic species have been described (Mathieu et al., 2011). The main form of seed dispersal occurs through resinous seeds that can stick to the feet, fur, and feathers of birds, bats, and insects (Valdebenito et al., 1990).

In popular medicine, *Peperomia* species have been used for the treatment of asthma, cough, ulcers, conjunctivitis, inflammation, and high cholesterol and have been shown to function as, diuretics, analgesics, and antibiotics (Wang et al., 2012). Crude extracts as well as isolated natural products from *Peperomia* species have also displayed numerous biological activities, including cytotoxicity (Wu et al., 2005, 2006), antifungal (Salazar et al., 2005), insecticide (Govindachari et al., 1998), anti-HIV (Zhang et al., 2007), anti-inflammatory (Li et al., 2007), and trypanocidal properties (Felippe et al., 2008). Despite this interest, only 38 species have been chemically studied, leading to the isolation of more than 200 compounds of different classes, and highlighting the great chemodiversity of this genus (Gutierrez et al., 2016). Among them, the most conspicuous classes of compounds are phenylpropanoids (Manalo et al., 1983; Gutierrez et al., 2016), tetrahydrofuran lignans (Felippe et al., 2008), secolignans (Monache and Compagnone, 1996), furofuran lignans (Mota et al., 2009), flavonoids (Gutierrez et al., 2016), and amides (Salazar et al., 2012). The species *Peperomia pellucida*, popularly known as “little heart,” “frog tongue,” “glass herb” and “jabuti herb” (Arrigoni-Blank et al., 2004), is the most chemically studied *Peperomia* species due to its worldwide distribution and various applications in traditional medicine (Majumder, 2011; Ooi et al., 2012; Sussa et al., 2013; Olabanji et al., 2014).

Initial studies on the chemical composition of essential oil from leaves of *P. pellucida* revealed the presence of the phenylpropanoids dillapiol and apiol as major constituents, in addition to mono- and sesquiterpenes (Silva et al., 1999). While recently samples of roots from *P. pellucida* from Nigeria were shown to contain β-farnesene as a major compound in the essential oil (Usman and Ismaeel, 2020). These analyses complement numerous other phytochemical studies of *P. pellucida* which confirmed the occurrence of apiol and dillapiol (Manalo et al., 1983; Rojas-Martínez et al., 2013), tetrahydrofuran lignans, secolignans, furofuran lignans (Xu et al., 2006), flavonoids (Aqil et al., 1993; Pappachen and Chacko, 2013), aurantiamide acetate (Ragasa et al., 1998), and chromenes in this species (Susilawati et al., 2015). Among them, pellucidin A, without configuration assigned, was formerly isolated from the stem bark of *Pachypodanthium staudtii* (Anonnaceae) (Ngadjui et al., 1989) and called pachypophyllin. Almost 10 years later, the same compound was also isolated from the leaves of *P. pellucida* (Ragasa et al., 1998), and soon later, the compound was proposed to have the *cis* configuration between the aromatic rings and was named pellucidin A (Bayma et al., 2000). However, by a combination of synthesis, ^1^H and ^13^C NMR data and X-ray analysis, its stereochemistry was revised to the *trans-*configuration, matching with all previously reported data (Riener and Nicewicz, 2013). Recently, pellucidin A was demonstrated to have anti-inflammatory and antinociceptive effects with a potential mechanism involving interaction with the nitric oxide pathway (Santos Queiroz et al., 2020).

From a biosynthetic point of view, pellucidin A can be rationalized as a dimer of styrene and formally considered a dinor-lignan, assuming decarboxylation steps of the cinnamic acid dimer linked by β − β carbons (Moss, 2000). The presence of cyclobutane rings in lignans, neolignans, and norlignans is relatively frequent, with several cases described (Kikuchi et al., 1983; Badheka et al., 1987; Malhotra et al., 1990; Takeda et al., 1990; Wang et al., 2000; Cuong et al., 2001; Davis et al., 2007; Li et al., 2007; Riener and Nicewicz, 2013). The biosynthesis of these cyclobutane rings in plants is predicted to result from a [2+2] cycloaddition reaction, which occurs upon irradiation with light (Tan et al., 2006; Bach and Hehn, 2011; Liu et al., 2011; Zhang et al., 2013; Zhao et al., 2013; Hong and Tantillo, 2014; Pan et al., 2015; Tang et al., 2015). However, an alternative mechanism for the biosynthesis of these cyclobutanes has been proposed to occur through enzyme-mediated reactions (Hao et al., 2001). Theoretical studies of this dimerization have been carried out with the aim of deciphering the mechanisms involved in the formation of cyclobutanes (Ortuno et al., 2005; Rappoport and Liebman, 2005; Hoffmann, 2008; Bach and Hehn, 2011; Hong and Tantillo, 2014). The biosynthetic study of (*E*)- and (*Z*)-hinokiresinol isomers for instance describes their formation from the oxidative coupling between a *p*-coumaryl alcohol and a *p*-coumaric acid unit, followed by a rearrangement and decarboxylation of the cinnamic acid moiety (Suzuki et al., 2001; Suzuki and Umezawa, 2007). In the case of pellucidin A, one of the possibilities for the formation of the cyclobutane ring involves a dimerization between two units of 2,4,5-trimethoxystyrene through a [2+2] cycloaddition. This hypothesis is supported by the co-occurrence of pellucidin A and 2,4,5-trimethoxystyrene in both *P. staudtii* and *P. pellucida* (Ngadjui et al., 1989). However, the oxidative coupling of two 2,4,5-trihydroxystyrene units, followed by a series of methylations to form pellucidin A, could also be considered a possible biosynthetic pathway (Bayma et al., 2000). Indeed, few studies have been carried out to investigate the precise sequence of intermediates and mechanisms involved in its formation. Herein, we report comprehensive phytochemical studies and biosynthetic studies with ^13^C-labeled precursors to describe the events leading to the formation of pellucidin A.

## Materials and Methods

### Reagents

Ethyl acetate (EtOAc), hexane, chloroform (CHCl_3_) and formic acid were purchased from LabSynth (SP, Brazil). Methanol (MeOH) and acetonitrile (ACN) (HPLC grade) were obtained from J.T. Baker (United States). All solvents were distilled prior to use. The deuterated chloroform used in ^1^H NMR analyses and (2-^13^C)-malonic acid and (^13^C)-methane iodine were purchased from Sigma-Aldrich.

All reactions were performed in oven dried glass (temperature ≥100°C) and cooled in a desiccator. Tetrahydrofuran (THF) was refluxed and distilled with sodium/benzophenone under a N_2_ atmosphere, and triethylamine and pyridine were refluxed and distilled from calcium hydride under a N_2_ atmosphere.

Analytical grade solvents (Merck^®^, Tedia^®^, and J.T. Baker^®^) and water purified using a Milli-Q system (Millipore^®^) were used in the chromatographic and spectroscopic analyses. High purity, commercially obtained reagents, were used without prior purification.

### Equipments

Chromatographic analyses were performed on a Shimadzu model SCL-10AVP apparatus equipped with two LC-10AD analytical pumps connected to an SPD-M10AVP diode drag detector and an SIL-9A automatic injector controlled by a communication module SCL-10AVP. The analyses were performed on a Phenomenex^®^ reverse phase C-18 column (Luna C-18 150 × 4.6 mm, 5 μm), and the data were analyzed using the program Class-VP version 6.10 program. All samples were dissolved in methanol (HPLC grade) at a concentration of 1 mg/mL and filtered through a 0.45 μm filter (Acrodisc CRPTFE). The injection volume was 20 μL. HPLC elution was performed using a gradient of solvents A (H_2_O + 0.01% formic acid) and B (ACN + 0.01% formic acid): 0–2 min (A:B, 7:3); 10 min (3:2); 45 min (0:1); 50 min (0:1); 55 min (7:3).

The extracts from the incorporations were analyzed by HPLC-MS using a Shimadzu SPD-M10AVP diode array detector. The data were then analyzed using the program Class-VP version 6.10 and mass spectrometric analyses were performed on a Bruker, Esquire 2000 plus in positive electrospray mode, 4.5 kV capillary voltage and 40 eV in the skimmer.

GC-MS analyses were performed using a Shimadzu GCMS-QP2010 Ultra equipped with a high AOC-5000 Plus injector. The system was operated in electron ionization (EI) mode at 70 eV on a Rxi^®^-5 ms (Crossbond^®^ 5% diphenyl/95% dimethylpolysiloxane) column of 10 m × 0.10 mm ID × 10 μm df. The injection temperature was 250°C, and the samples were eluted on a programmed ramp of 40–280°C at a rate of 25°C/min. Helium was used as the carrier gas at a rate of 0.56 mL/min in split mode (1:30).

^1^H and ^13^C NMR analyses were carried out using in Bruker DPX 300 (300 MHz ^1^H NMR, 75 MHz ^13^C NMR) or a Bruker DRX 500 (500 MHz ^1^H and 125 MHz ^13^C) NMR. The values of chemical shifts (δ) were shown in ppm, and J were given in Hertz.

#### Analytical and Preparative Planar Chromatography

The TLC separations were performed on Merck^®^ plates, silica gel 60, with fluorescence indicator PF_254_ and an aluminum support of thickness 0.2 mm. The prep-TLC was carried out on 20 × 20 cm size glass plates, 1.0 mm thickness of Merck silica gel 60 and fluorescence indicator PF_254_. The plates were visualized under 254 and 365 nm ultraviolet light or nebulized with sulfuric vanillin solution followed by heating.

For circular chromatography, round glass plates coated with a 2.0 mm thick layer of silica gel 60, PF_254_ containing gypsum, were used. The compounds were separated using hexane-EtOAc as the eluant, and were latter visualized under ultraviolet light (254 and 365 nm).

Purification of compounds by column chromatography was performed using VLC (vacuum liquid chromatography), with columns of appropriate length and diameter necessary for the masses of the samples (Pelletier et al., 1986). Silica gel 60 HF_254_ (70–230 mesh ASTM) from Merck^®^ and C-18 reverse phase silica were used as the stationary phase. The ratio of silica used to pack the column was approximately 20 times the mass of the sample to be purified.

#### Plant Material and Insects

The specimens of *P. pellucida* were maintained in the greenhouse facilities of the Institute of Chemistry at the University of São Paulo (IQUSP) under controlled conditions (25 ± 2°C; photoperiod of 12 h; 85 W fluorescent lamps). The *Piper* and *Peperomia* species growing in the garden at IQUSP naturally host several arthropods, such as *Edessa meditabunda* (Hemiptera), *Monoplatus* sp. (under identification; Coleoptera; Chrysomelidae), and *Geometridae* sp. (under identification, Lepidoptera) that were collected in the garden and used in biotic plant stress experiments (Supplementary Figure 1). The experiments with herbivores wounding were carried out in cages using five replicates containing three plants with three herbivores each, excepting for Geometridae, which had two caterpillars. The time-length varied among herbivores because of the different rate of plant consumption. While for *Monoplatus* sp. and *E. meditabunda* the experiment took 7 days, for the voracious Geometridae caterpillars the plants were collected after 2 days. For the drought treatment the plants were kept for 7 days until the soil became dry while in the case of the high temperature treatment, the plants were maintained at 40°C for 7 days. For the dark treatment, the plants had the photoperiod turned off for 7 days or until the plants showed a sign of a loss of chlorophyll (4–5 days). After each treatment using five replicates, the plants were individually extracted, and the crude CHCl_3_ fractions were submitted to ^1^H NMR analysis. The resulting data was then subjected to a multivariate analysis.

#### Preparation of Crude Extracts for Metabolome Analysis

The leaves of the mature plants (approximately 6 months old, when plants start to produce seeds) and seedlings (1–4 months old) were frozen immediately after sampling using liquid N_2_ and crushed in a mortar and pestle until a fine powder was obtained. A total of 100 mg of ground plant material was then transferred to a 2 mL Eppendorf tube and vortexed for 5 min with a mixture of solvents (1 mL; chloroform:methanol:water; 2:1:1; v/v/v). The samples were then centrifuged at 10,000 rpm at 0°C for 10 min, and organic phases were collected using a pipette. The extraction procedure was repeated, and the pooled chloroform fraction was dried under a N_2_ stream, while the aqueous extract was dried using a SpeedVac (vacuum centrifuge). The resulting samples were stored at −20°C until analysis.

#### Fractionation and Purification of Secondary Metabolites

Fresh aerial parts of *P. pellucida* (500 g) obtained from cultivated plants were extracted using the same procedure as above and after concentration using a rotary vaporator, 4.12 g of crude CHCl_3_ fraction was obtained. This extract was suspended in MeOH:H_2_O (1:4, v/v, 400 mL) and subjected to a dechlorophylation step in a Celite column as described (Fernandes et al., 1997), yielding a chlorophyll-free fraction (1.12 g). This fraction (1.00 g) was then separated with a VLC system using silica gel, eluted with a gradient of hexanes:EtOAc (9:1 – 0:1) and then with EtOAc:MeOH (9:1 – 0:1), yielding 95 fractions (10 mL each). Similar sub-fractions were pooled (up to 15 fractions) based on TLC analysis. The main compounds in the fractions were purified by circular chromatography (Chromatotron^®^) eluted with a gradient of hexanes:EtOAc (9:1 – 0:1) yielding 2,4,5-trimethoxycinnamic acid (**1**, 6.0 mg), 2,4,5-trimethoxystyrene (**2**, 14.1 mg), 2,4,5-trimethoxybenzaldehyde (**3**, 19.7 mg), dillapiol (**4**, 45.8 mg), pellucidin A (**5**, 3.4 mg), sesamin (**6**, 15.3 mg), and 5,6,7-trimethoxyflavone (**7**, 121.3 mg).

#### Calibration Curve

Pellucidin A (5), 5,6,7-trimethoxyflavone (**7**), sesamin (**6**), 2,4,5-trimethoxystyrene (**2**), 2,4,5-trimethoxycinnamic acid (**1**), 2,4,5-trimethoxybenzaldehyde (**3**), and dillapiol (**4**) were quantified by HPLC-DAD. Calibration curves were generated using standard solutions of the respective purified compounds. A stock solution in MeOH (1 mg/mL) was prepared for each compound and diluted to 1.000, 0.5000, 0.2500, 0.125, 0.0625 and 0.0312 mg/mL. All of the solutions were analyzed by HPLC under the same conditions of analysis as the extracts.

#### Pellucidin A Formation During the Extraction Process

Despite the diverse reports of natural compounds containing cyclobutane rings, some authors claim that these compounds could be formed during the extraction process (Seidel et al., 2000; McCracken et al., 2012). Thus, the dimerization of 2,4,5-trimethoxystyrene to pellucidin A was evaluated during the extraction procedures of leaves of *P. pellucida.* In two sets of extractions, 1 mg of ^13^C-natural abundance 2,4,5-trimethoxystyrene or (8-^13^C)-2,4,5-trimethoxystyrene solutions in CHCl_3_ (500 μL) were spiked in the extraction procedures, using the protocol for metabolome analysis. The formation of pellucidin A under such conditions was evaluated by comparison of relative intensities of molecular ions by GC-MS analysis, in which the abundance of [M+1]^+.^ and [M+2]^+.^ provide data for incorporation of one or two unities of ^13^C-labeled precursors (Opitz et al., 2014). The data were compared with the control experiments without spiking either natural or labeled 2,4,5-trimethoxystyrenes.

#### Multivariate Analysis of NMR Data of Plants

An amount of 5–7 mg of the crude extracts dissolved in CDCl_3_ was submitted to ^1^H NMR analysis at 300 MHz, and the data were processed using the program MestreNova. The scale was adjusted with the TMS signal as 0.00 ppm, the range of chemical shift data was between 1.4 and 12 ppm, and the residual chloroform signal between 7.20 and 7.28 ppm was discarded. A binning was made for a range of 0.02 ppm, and the data were normalized by the total area. The data were saved as an ASCII text file and processed by The Unscrambler program (version 9.5, CAMO Process AS, Norway).

#### Preparation of Cinnamic Acids

To investigate the substrate specificity in the formation of pellucidin A in *P. pellucida*, a series of synthetic ^13^C-labeled cinnamic acids and styrenes were evaluated by *in vivo* feedings and enzymatic conversion experiments. The cinnamic acids were synthesized by a Knoevenagel condensation between malonic acid and benzaldehyde derivatives. Malonic acid (9.60 mmol) and the benzaldehydes (10.00 mmol) were dissolved in pyridine (3.5 mL), followed by addition of 10 μL of piperidine and 10 μL of aniline, both previously distilled, as described (Katayama et al., 1992). After the reaction was completed, the mixture submitted to work-out yielded colorless crystals of cinnamic acids (Supplementary Figure 9; A1–A16) with a typical yield above 90%. The structures of the cinnamic acids were characterized using ^1^H and ^13^C NMR and EIMS data in addition to a comparison with previously published data (Katayama et al., 1992; Mitra and Karchaudhuri, 1999). For the preparation of isotopically labeled versions of cinnamic acids, (2-^13^C)-malonic acid (99%) from Sigma-Aldrich was used and the corresponding cinnamic acids were purified and characterized spectroscopically.

#### Preparation of Methyl Triphenylphosphonium Iodides

Into a 50 mL two-necked flask under reflux was added 125 μL (2 mmol) of methyl iodide and 0.84 g (3.2 mmol) of triphenylphosphine in 30 mL of dry tetrahydrofuran. The reaction was kept for 12 h at a temperature of 110°C. Then, the white solid formed was washed with hexane (3 × 50 mL) and vacuum dried (Sato et al., 2005).

#### Preparation of Styrenes

The styrenes were prepared via the Wittig reaction using the respective benzaldehydes and the methyl triphenylphosphonium iodide prepared above (Simpson et al., 2005; Farina et al., 2007). Then, 3 mL of dry tetrahydrofuran and 1.2 mmol methyltriphenylphosphonium iodide were added into a 25 ml three-necked flask equipped with magnetic stirring. The mixture was stirred at −40°C, and after 20 min, 1 mL (12.85 mmol) of *n*-BuLi was added, after which the slightly yellow solution was kept in an ice bath at 0°C under stirring for 30 min. Following that, a solution of 1.2 mmol of benzaldehydes in 2 ml of dry THF was added over approximately 30 min and stirred for 10 h at room temperature. The solution was then poured into 50 mL of ice water and extracted with hexanes (3 × 50 mL), dried and concentrated to dryness. The crude extract was then subjected to a 4 cm flash silica column using hexanes:EtOAc (9:1; v/v) as the mobile phase, yielding the styrenes (Supplementary Figure 10; S1–S16) and two hydrogenated derivatives (S17 and S18). For the preparation of the isotopically labeled styrenes, the methyl triphenylphosphonium iodide was prepared from ^13^CH_3_I.

The preparation of the phenolic styrenes was performed starting from the demethylation of the respective methoxy-benzaldehydes using BBr_3_ (Unver et al., 2011). To a solution of 1.2 mmol of the methoxy benzaldehydes in chloroform (12.5 mL) was added 0.306 mmol (2 eq) BBr_3_ at 0°C. The reaction mixtures were stirred under a N_2_ stream for 12 h. After that, a solution of 25 mL of sodium hydroxide was added. Then, concentrated H_2_SO_4_ was added, and the precipitate was extracted with ethyl ether. The organic fraction was dried by anhydrous Na_2_SO_4_, filtered, and concentrated under vacuum using a rotary evaporator.

Hydroxybenzaldehydes (2.40 mmol), *tert*-butyldimethylsilane chloride (2.88 mmol), and imidazole (4.8 mmol) were added to a 50 mL round bottom flask and were heated in a microwave at 90 W (2 min) and then to 180 W (3 min). The reaction was quenched by the addition of 15 mL of water, followed by extraction with ethyl ether. The organic phases were combined, dried with anhydrous Na_2_SO_4_ and evaporated under reduced pressure. The crude product was purified in a short VLC column eluted with hexanes:EtOAct (7:3; v/v), with typical yield of the reactions being 80%. All protected compounds were characterized by GC-MS analysis (Bastos et al., 2005).

The TBS-protected phenolic styrenes (0.898 mmol) were dissolved in 3.0 mL of dry THF. The solution was cooled to 0°C in an ice bath while constantly being stirred for 30 min. Then, a solution of 312 μL of tetrabutylammonium fluoride (TBAF) was added slowly to 1.2 mol/L in dry THF, while the mixture was stirred for 30 min. The crude products were purified by VLC eluted with hexanes:EtOAct (4:1; v/v), and a total of 16 styrenes were subsequently isolated and characterized by GC-MS and ^1^H and ^13^C NMR analysis. The hydrogenated derivatives (S17 and S18) were obtained by treatment of corresponding styrenes (S1 and S10) with H_2_-Pd/C in dichloromethane at room temperature overnight.

#### *In vivo* Administration of the Precursors in *P. pellucida*

The leaves of *P. pellucida* (3–4 months old) were cultivated for 21 days in a hydroponic system. After this period, the leaves with root formation were transferred to Eppendorf tubes containing a solution of isotopically labeled precursors in pure water and kept for various periods of time. Specific details for the incorporation of each substrate are provided in the following sections.

#### Administration of the Labeled Precursor L-(2-^13^C)-Phenylalanine

Solutions containing 1.5, 3.0, and 6.0 μmol of L-(2-^13^C)-phenylalanine in 100 μL of Milli-Q water were used. Aliquots of 1.0 mL of the different concentrations of the isotopically labeled precursors were administered in the hydroponic system of the plants maintained in Eppendorf tubes. The administration periods of the isotopically labeled precursors in the plants were 12, 24, 48, 72, 96, and 120 h at 25°C, with replenishment of the precursor solutions when needed. All experiments were carried out in triplicate and the blank control was administered water with DMSO (0.5%) with no labeled precursor added.

The leaf samples were then frozen in liquid nitrogen and extracted with CHCl_3_:MeOH:H_2_O (2:1:1; v/v/v). The chloroform fraction was separated, and the solvent was evaporated under vacuum. The samples were resuspended in MeOH filtered through a 0.45 μm membrane and subjected to GC-MS analysis. The quantification of the precursor incorporations was performed as described (Opitz et al., 2014).

#### Administration of (8-^13^C)-Cinnamic Acids and (8-^13^C)-Styrenes

The administration of (8-^13^C)-cinnamic acids (A1–A16) and (8-^13^C)-styrenes (S1–S18) in plantlets of *P. pellucida* was performed under the same conditions as for L-(8-^13^C)-phenylalanine. The (8-^13^C)-cinnamic acids were solubilized in 0.5 μl of DMSO and brought to 1 mL with water. Each of the concentrations, 1.0, 2.1, and 4.2 μmol of the cinnamic acids in 100 μL of the solution, were fed for the same intervals as in the above section. The plant material was then treated, the crude extracts analyzed by GC-MS, and the incorporation of the precursors was quantified as above (Opitz et al., 2014).

#### Obtention of Enzymatic Extract and Assays

Leaves crushed using liquid N_2_ were extracted with 100 mL of pH 7.0 potassium phosphate buffer and EDTA (0.12 mmol), ascorbic acid (0.2 mmol), MgCl_2_ (0.31 mmol), and polyvinylpolypyrrolidone (45.0 mmol) under gentle stirring for 30 min at 4°C followed by filtration with miracloth tissue. An aliquot of the resulting supernatant was separated, and the precipitate was discarded. Saline precipitation was performed using the soluble fraction from the first centrifugation, slowly adding 1.0 mmol of (NH_4_)_2_SO_4_, under constant stirring. The solution was centrifuged for 10 min at 13,000 rpm, yielding a pellet that was used as the protein source for the enzymatic assays. The protein contents were determined as 33.3 ± 3.7 mg/mL (Bradford, 1976). The enzymatic conversions were performed using 100 μL of protein extract, 20 μL of the substrate solutions at 10 mM and 76 μL of potassium phosphate buffer at 40 mmol.L^–1^ (pH 7.0), as described (Vassao et al., 2006). The reaction mixtures were incubated for 30, 60, 90, or 120 min at 25°C, after which they were terminated by extraction with EtOAc (2 × 500 μL). The blank control had only substrates without proteins or only protein preparations using the same incubation buffer. To verify a possible oxidative coupling reaction, H_2_O_2_ and 50 mM NADPH were tested as oxidants both together and separately (Lu et al., 2004; Vassao et al., 2006). The enzymatic fractions, precursors and crude reaction products were analyzed by HPLC, using a Phenomenex^®^ reverse phase C-18 column (Luna C-18 150 × 4.6 mm, 5 μm), with a flow of 1 mL.min^–1^, and a detector set at λ = 260 nm.

## Results and Discussion

### Phytochemical Study of *P. pellucida*

**FIGURE 1:**
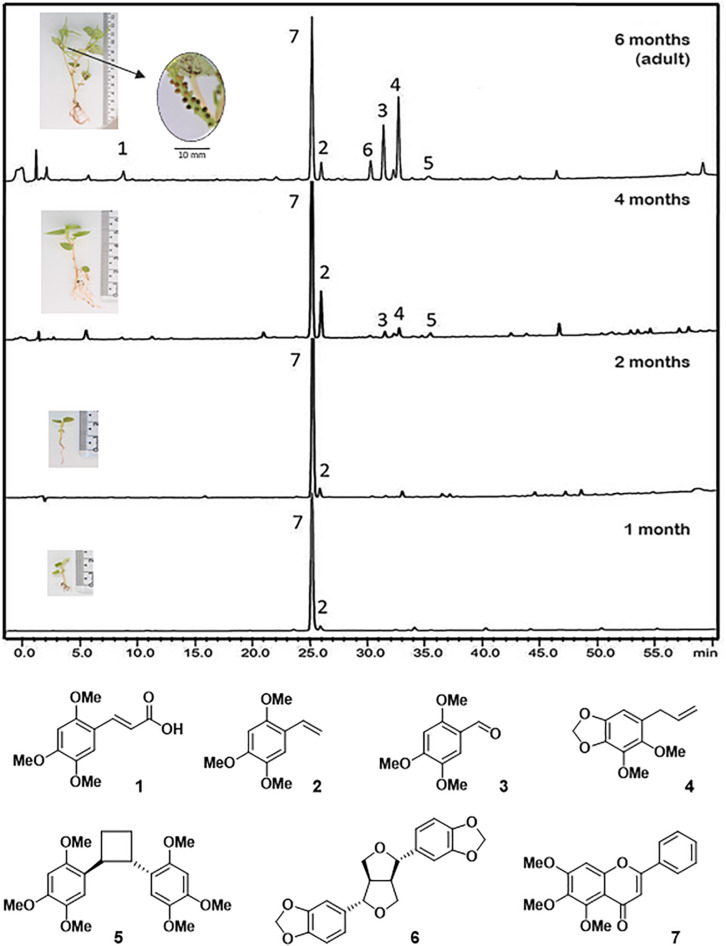
Chromatographic profile (HPLC) of crude extracts of leaves at different life stages of *P. pellucida*. 2,4,5-trimethoxycinnamic acid (**1**); 2,4,5-trimethoxystyrene (**2**); 2,4,5-trimethoxybenzaldehyde (**3**); dillapiol (**4**) of pellucidin A (**5**); sesamin (**6**); and 5,6,7-trimethoxyflavone (**7**). The detection wavelength was set at 260 nm. Average height for plants is shown in the boxes: 10–20 mm (1 month); 20–30 mm (2 months); 40–50 mm (4 months); 50–70 mm (6 months); at 6 months the plants start to produce seeds (<1 mm; see magnification) and are considered adults.

The isolation of pellucidin A was from the aerial parts of *P. pellucida* (Ragasa et al., 1998; Bayma et al., 2000), but formerly it was isolated from *P. staudtii* (Anonnaceae) (Ngadjui et al., 1989). In this work we detected pellucidin A as minor compound based on HPLC-PDA and GC-MS data from crude leaves extracts. Moreover, we detected additional compounds not previously identified from this species. Then, a large-scale crude extract from leaves was submitted to chromatographic fractionation to isolate these compounds and to obtain a standard of pellucidin A. In total, seven compounds were isolated and characterized as 2,4,5-trimethoxycinnamic acid (**1**), 2,4,5-trimethoxystyrene (**2**), 2,4,5-trimethoxybenzaldehyde (**3**), dillapiol (**4**), pellucidin A (**5**), sesamin (**6**), and, 5,6,7-trimethoxyflavone (**7**) (Figure 1, Table 1, and see Supplementary Figures for mass and NMR spectra).

**TABLE 1:** Content of secondary compounds in different parts of *P. pellucida* (mg/g extract ± SD).

**Compounds**	**Leaves**	**Stems**	**Roots**	**Fruits**
2,4,5-trimethoxycinnamic acid (**1**)	9.7 ± 0.99	–	–	–
2,4,5-trimethoxystyrene (**2**)	17.7 ± 1.21	16.3 ± 0.76	35.2 ± 0.88	26.3 ± 1.80
2,4,5-trimethoxybenzaldehyde (**3**)	19.0 ± 0.89	24.5 ± 0.95	–	–
Dillapiol (**4**)	48.9 ± 1.52	27.3 ± 1.61	–	4.4 ± 0.45
Pellucidin A (**5**)	3.5 ± 0.39	2.3 ± 0.23	–	–
Sesamin (**6**)	22.5 ± 3.21	–	60.6 ± 2.31	–
5,6,7-trimethoxyflavone (**7**)	126.5 ± 8.32	138.0 ± 5.45	102.0 ± 1.0	99.8 ± 3.5

Pellucidin A (**5**) was identified by analysis of ^1^H and ^13^C NMR data (Supplementary Figures 24, 25), and mass spectral data (ESI-TOF and MS-IE; Supplementary Figures 22, 23) as well as by comparison with reported data (Bayma et al., 2000; Riener and Nicewicz, 2013). The structure of a head-to-head coupling mode to pellucidin A based on the analysis of ^1^H and ^13^C NMR data fully agreed with the *trans* configuration described for it (Riener and Nicewicz, 2013).

Compound **1** was identified as 2,4,5-trimethoxycinnamic acid based on ^1^H and ^13^C NMR data (Supplementary Figures 12, 13), and mass spectral data (MS-EI) (Supplementary Figure 11) as well as by comparison with reported data (Sinha et al., 2003). Compound **2** was identified as 2,4,5-trimethoxystyrene by analysis of ^1^H and ^13^C NMR data (Supplementary Figures 15, 16) and mass spectral data (MS-EI; Supplementary Figure 14) as well as by data reported in the literature (Manalo et al., 1983). Compound **3** was purified as a white solid and identified as 2,4,5-trimethoxybenzaldehyde by analysis of ^1^H and ^13^C NMR data (Supplementary Figures 18, 19) and mass spectral data (MS-EI; Supplementary Figure 17; Li et al., 2012). Dillapiol (**4**) was identified by analysis of ^1^H NMR data (Supplementary Figure 21) and mass spectrum data (MS-IE; Supplementary Figure 20) (Bayma et al., 2000; Chan, 2014). Compound **6** was identified as furofuran lignan sesamin by analysis of ^1^H and ^13^C NMR data (Supplementary Figures 27, 28) and mass spectral (MS-EI; Supplementary Figure 26) as well as by comparison with data previously described for this compound (Fukuda et al., 1986). Finally, the major compound **7** present in all organs of *P. pellucida* was identified as 5,6,7-trimethoxyflavone (Bernini et al., 2008). The flavone **7** was purified as a yellow solid and identified using ^1^H and ^13^C NMR data (Supplementary Figures 31, 32) and mass spectral data (HRESIMS and MS-EI; Supplementary Figures 29, 30).

The isolated compounds from *P. pellucida* are represented according to their biosynthetic relationship (Figure 4). While the major compound flavone (**7**) has no oxygenation in the aromatic ring B (pendant ring), separate biosynthetic pathways would produce the lignan sesamin (**6**), which was shown to be biosynthesized in sesame seeds (*Sesamum indicum*) by oxidative coupling of coniferyl alcohol, which has two guaiacyl aromatic ring (4-hydroxy-3-methoxyphenyl), such as in the structure of ferulic acid, forming initially the lignan pinoresinol and then followed by two methylenedioxy bridge formation to produce the lignan sesamin (Kato et al., 1998). The three compounds 2,4,5-trimethoxycinnamic acid (**1**), 2,4,5-trimethoxystyrene (**2**), and 2,4,5-trimethoxybenzaldehyde (**3**) have the same oxygenation pattern as pellucidin A. Therefore, it is plausible to suggest their biosynthetic relationship. The 2,4,5-trimethoxybenzaldehyde (**3**), formerly found in the leaves of *Peperomia tetraphylla* (Li et al., 2012), is described here for the first time in *P. pellucida*, and it could be produced by oxidative cleavage of the 2,4,5-trimethoxystyrene (**2**).

### Quantification of Compounds in Extracts

The quantification of the compounds isolated from the different parts of *P. pellucida* was determined according to the methodology described (Collins et al., 1995). The 5,6,7-trimethoxyflavone (**7**) is the major secondary metabolite in all parts of the plant (Table 1). The phytochemical analysis allowed the characterization of pellucidin A in the leaves of *P. pellucida*, which was also detected in the stems and fruits of the plant but only as a minor compound.

### Chemical Profile of *P. pellucida* During Ontogeny

The monitoring of the content of secondary compounds in *P. pellucida* during ontogeny was carried out by analysis of leaflets from 1-, 2-, 4-, and 6-months old plants. At 6 months, the plants begin to produce fruits and viable seeds and are considered adults. While the crude extracts from the leaves were subjected to ^1^H NMR analysis and principal component analysis (PCA), the identification of the compounds was carried out by GC-MS and HPLC analysis of the crude extracts aided with comparison with pure compounds (Figure 1).

**FIGURE 2:**
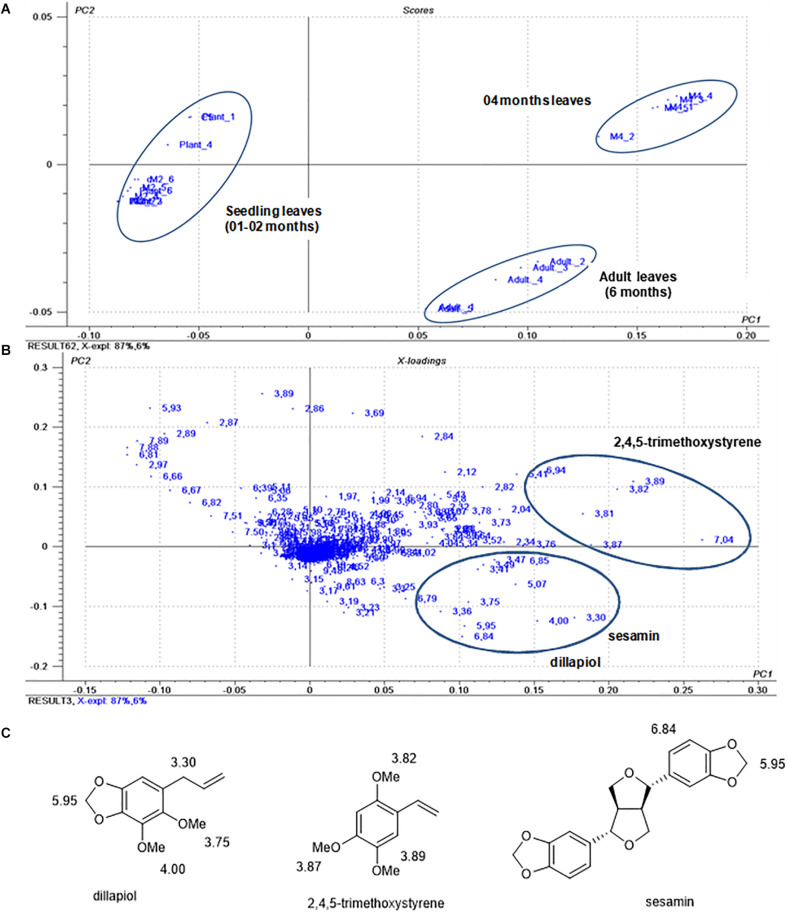
Score plots **(A)** and loadings **(B)** of the principal component analysis of *P. pellucida* samples during ontogeny. **(C)** Structures of dillapiol (**4**), 2,4,5-trimethoxystyrene (**2**) sesamin (**6**) with assignment of chemical shifts in the ^1^H NMR spectra.

In the PCA analysis using ^1^H NMR data from the crude extracts, the score plot (Figures 2A,B) showed 93% of the variance between the data, 87% of which were explained by PC1 and 6% by PC2. A clear discrimination between seedlings and adult leaves was observed. In the loading plots (Figure 2), the main variables responsible for the discrimination of the groups were assigned to the chemical shifts of methoxy groups (δ 4.00 and 3.75), benzylic methylene (δ 3.30) and of methylenedioxy group (δ 5.95) of dillapiol and of sesamin and of aromatic hydrogens (δ 6.84) of sesamin (Figure 2C). Another cluster observed in the PCA analysis corresponded to the 4-month-old samples in which the chemical shifts of 2,4,5-trimethoxystyrene at δ 3.82, 3.87 and 3.89 associated with the methoxy groups and at δ 7.04 relative to the vinyl group were evidenced (Figures 2A–C).

HPLC-UV analysis of crude extracts from *P. pellucida* leaves at different developmental stages showed that 5,6,7-trimethoxyflavone (**7)** is the major constituent throughout the stages with clear dominance at early seedling stages (Figure 1; 1–2 months old). At the 4th month, 2,4,5-trimethoxystyrene (**2**), dillapiole (**4**), pellucidin A (**5**), and sesamin (**6**) appeared in detectable amounts and then, at the adult phase (6 months) the compounds **1**, **3**, **4**, and **6** had their relative content significantly increased (Figure 1).

Several studies have shown changes in the content of secondary metabolites in seedlings that are assumed to provide some level of defense against natural enemies (Barton, 2008; Elger et al., 2009; Cirak et al., 2013; Gaia et al., 2014). Quantitative and qualitative variations in the chemical profile of plants at different developmental stages after germination have already been reported for *Piper gaudichaudianum*, in which dillapiole and apiol are initially the main compounds and then are replaced by gaudichaudianic acid throughout development (Gaia et al., 2014). Similar studies were conducted with *Cannabis sativa* (Vogelmann et al., 1988), *Virola sebifera* (Danelutte et al., 2000), and *Araucaria angustifolia* (Fonseca et al., 2000). In the case of *P. pellucida*, the production of 5,6,7-trimethoxyflavone would provide photoprotection at an early developmental stage, and with the biosynthetic apparatus directed toward growth instead of producing the entire pathways leading to pellucidin A (Boege and Marquis, 2005).

### Chemical Profile of *P. pellucida* Leaves Under Treatments

**FIGURE 3:**
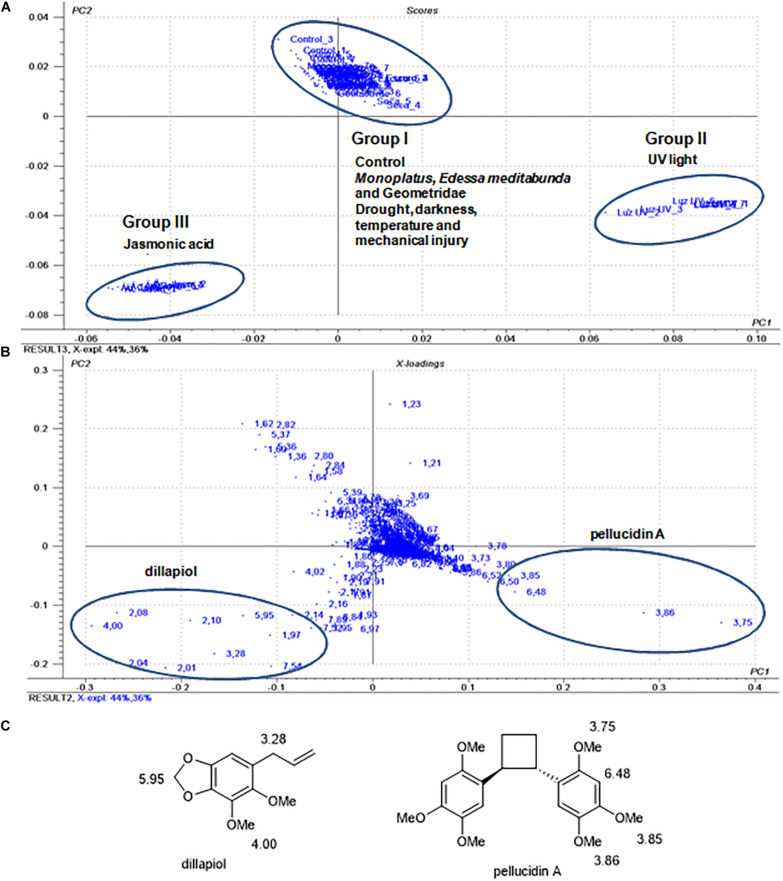
Score plot **(A)** and loadings **(B)** of principal component analysis of ^1^H NMR data from *P. pellucida* samples (under different treatments). **(C)** Structure of pellucidin A (**5**) and assignment of chemical shifts in the ^1^H NMR spectra found in the PCA loading plot of the *P. pellucida* samples.

Considering the low production of pellucidin A in the leaves of *P. pellucida*, a series of studies was conducted to determine whether the effect of different stress conditions would affect its chemical profile. Thus, in addition to the control plants, treatments included mechanical injury, drought stress, exposure to UV_365_ light, darkness, high temperature (40°C) and treatment with jasmonic acid. The plant was also exposed to damage caused by herbivores such as *E. meditabunda* (Hemiptera), *Monoplatus* sp. (Coleoptera; Chrysomelidae), and an unknown Geometridae (Lepidoptera) (Supplementary Figure 1). These three arthropods have been observed naturally in an open garden at USP for several years. The specimens of *P. pellucida* were kept under the treatment conditions for 24, 48, 72, and 120 h. The leaves were then sampled and frozen in liquid nitrogen, the methanolic extracts analyzed by ^1^H NMR (300 MHz) and then the data submitted to PCA analysis (Figures 3A,B).

The PCA analysis of the samples (Figures 3A,B) had the main components (PC1 and PC2) accounting for 80% of the total data and with a discrimination of the samples in three distinct clusters. Group (I) contained the control and samples resulting from water stress, mechanical injuries, low light, darkness, temperature (40°C), and herbivory by adults of *E. meditabunda*, *Monoplatus* sp., and Geometridae caterpillars. This set of data indicated that these treatments did not cause detectable changes in the chemical profiles of the leaves. The HPLC-UV data (Supplementary Figure 2) were compatible with similarities in chemical profiles as compared to the control samples and were not further explored. However, the composition of leaf samples maintained under UV light (Group II) and treated with jasmonic acid (Group III) showed significant differences compared to the control (Group I). The analysis of loading plots of the samples resulting from the treatment with UV light (group II) indicated that the ^1^H NMR chemical shifts of pellucidin A (Figure 3C), specifically the signals assignable to methoxy groups at δ 3.75, 3.85 and 3.86 ppm and of the aromatic hydrogen H3-H3′ at δ 6.48, accounted for such discrimination.

Group III, corresponding to the plants treated with jasmonic acid, revealed chemical shifts at δ 5.95, 4.00, and 3.28 ppm relative to the methylenedioxy, methoxy, and allyl methylene groups, respectively, assignable to dillapiol as the main compound (Figure 3). HPLC analysis of leaves extracts of the samples resulting from treatment with jasmonic acid confirmed the increase in the production of dillapiol as compared to the control samples. Despite not being detected in the damaging experiments with herbivores, dillapiol is reported to be elicited after damage (Silva et al., 2012), and it is also known to have toxic and repellent properties against different insects (Bhuiyan et al., 2001; Araujo et al., 2012; Volpe et al., 2016).

The analysis of HPLC-UV and GC-MS of the samples of *P. pellucida* treated under UV light indicated an increase in the production of pellucidin A, similar to the previous case (Zhang et al., 2013; Zhao et al., 2013). Such an increase in the pellucidin A content indicates a potential photoprotective function in the plant similar to the role of 5,6,7-trimethoxyflavone. Interestingly, the same treatment with UV light applied to two other *Peperomia* species, *Peperomia glabela* and *Peperomia obtusifolia*, led these plants to decay after 48 h (data not shown).

### Incorporation of the Precursors Into Pellucidin A

#### Incorporation of L-(2-^13^C)-Phenylalanine

To elucidate the biosynthetic pathway of pellucidin A, preliminary feeding experiments were performed with L-(2-^13^C)-phenylalanine. The percentage of incorporation was determined by GC-MS analyses of the crude extracts of *P. pellucida* leaves incubated with isotopically labeled precursors compared to the control leaf extracts and pure pellucidin A (Tables 2, 3).

**TABLE 2:** Mass spectra data (molecular ions and % of relative abundance for [M+1]^+.^ and [M+2]^+.^) of compounds after incorporation of ^13^C-labeled precursors into 2,4,5-trimethoxycinnamic acid, 2,4,5-trimethoxystyrene, and pelludicin A in P. pellucida*.

**Precursor**	**2,4,5-trimethoxycinnamic acid**		**2,4,5-trimethoxystyrene**		**Pelludicin A**		
	**238 [M]^+.^**	**239 [M+1]^+.^**	**194 [M]^+.^**	**195 [M+1]^+.^**	**388 [M]^+.^**	**389 [M+1]^+.^**	**390 [M+2]^+.^**
L-(2-^13^C)-phenylalanine	100	22.6	100	45.1	0.23	0.13	0.03
(8-^13^C)-cinnamic acid	–	–	100	23.4	0.14	0.09	0.02
(8-^13^C)-ferulic acid	–	–	100	18.2	0.11	0.07	0.02
(8-^13^C)-2,4,5-trimethoxycinnamic acid	–	–	100	58.1	0.09	0.24	0.03
(8-^13^C)-2,4,5-trimethoxystyrene	–	–	–	–	0.06	0.10	0.17

*For comparison, only data obtained at 96 h are shown, excepting for (8-^13^C)-ferulic acid which was obtained at 48 h.

*For full mass spectra, see the corresponding Supplementary Figures as indicated in the Figure 5.*

**TABLE 3:** Percentage of incorporation of ^13^C-labeled precursors into pelludicin A in *P. pellucida*.

**Precursor**	**2,4,5-trimethoxycinnamic acid**	**2,4,5-trimethoxystyrene**	**Pelludicin A**
L-(2-^13^C)-phenylalanine	1.49 ± 0.10	3.92 ± 0.09	0.72 ± 0.03
(8-^13^C)-cinnamic acid	nd	2.89 ± 0.10	1.32 ± 0.11
(8-^13^C)-ferulic acid	0.04 ± 0.01	0.69 ± 0.03	0.51 ± 0.03
(8-^13^C)-2,4,5-trimethoxycinnamic acid	–	9.00 ± 0.14	7.50 ± 0.19
(8-^13^C)-2,4,5-trimethoxystyrene	–	–	12.80 ± 0.22

*For comparison data were obtained at 96 h of administration, except for (8-^13^C)-ferulic acid which was at 48 h. nd, not detected.

**FIGURE 4:**
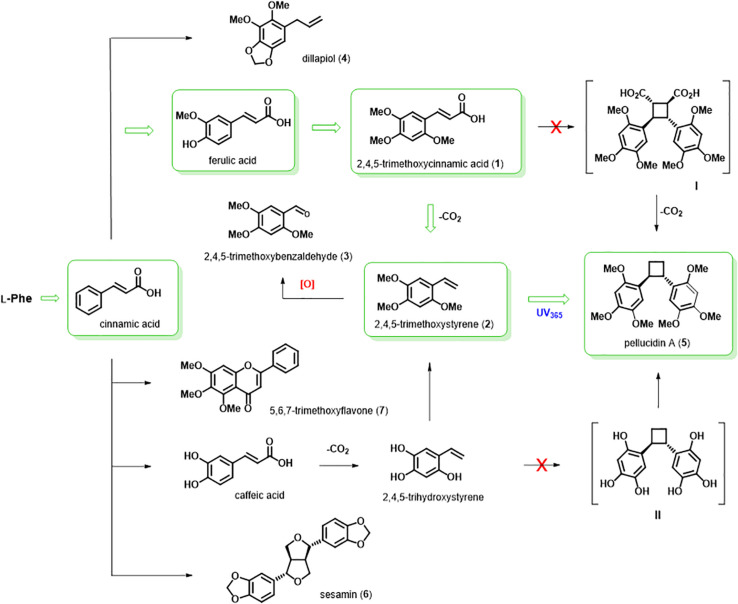
Proposed biosynthetic pathway for pellucidin A in *P. pellucida*. ^13^C-Labeled precursors fed and incorporated into pellucidin A (green arrow and boxes). The [2+2] photodimerization of 2,4,5-trimethoxystyrene using UV_365_ nm produce pellucidin A. Putative dimers I and II were not detected. Natural and isolated compounds are indicated by numbers (**1–7**) between parenthesis.

**FIGURE 5:**
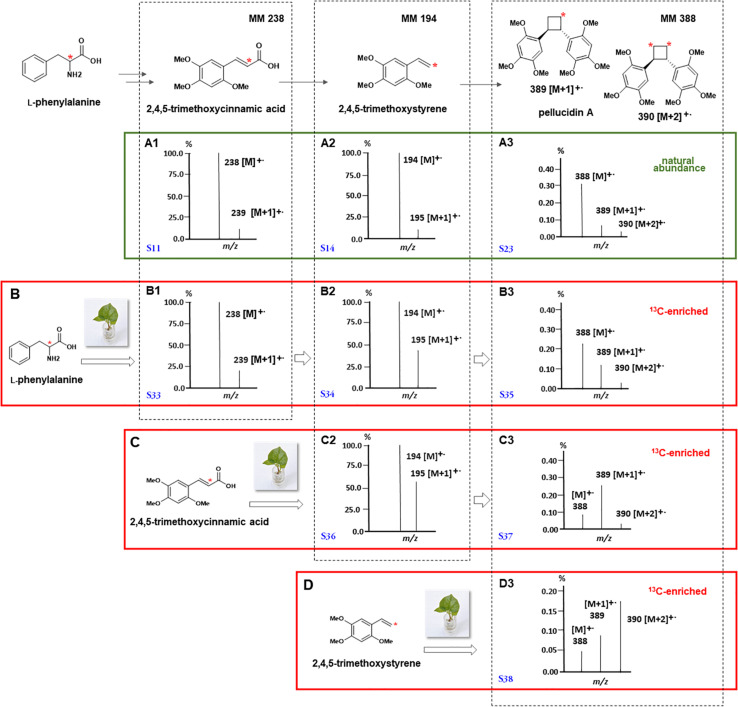
Mass spectra of the products formed by the incorporation of L-(2-^13^C)-phenylalanine **(B)**, (8-^13^C)-2,4,5-trimethoxycinnamic **(C)**, and (8-^13^C)-2,4,5-trimethoxystyrene **(D)** in *P. pellucida*. For comparison, mass spectra of 2,4,5-trimethoxycinnamic **(A1)**, 2,4,5-trimethoxystyrene **(A2)**, and pellucidin A **(A3)** at ^13^C-natural abundances are shown followed by the ^13^C-enriched compounds after administration of L-(2-^13^C)-phenylalanine **(B1–B3)**, (8-^13^C)-2,4,5-trimethoxycinnamic **(C2,C3)**, and (8-^13^C)-2,4,5-trimethoxystyrene **(D3)**. The regions of molecular ions for spectra of ^13^C enriched pellucidin A **(B3,C3,D3)** are highlighted together with their respective structures to show the relative intensities of the ions *m/z* [M+1]^+.^ and [M+2]^+.^ as results of the ^13^C enrichments. The mass spectra were reconstructed from the original data and the corresponding spectra can be found as Supplementary Figures 11–38. * Represents ^13^C.

First, considering the biosynthetic possibilities for pellucidin A formation (Figure 4), accurate analysis of the mass spectra of putative intermediates after feeding L-(2-^13^C)-phenylalanine indicated significant enrichment of 1.49 and 3.92% to 2,4,5-trimethoxycinnamic acid and 2,4,5-trimethoxystyrene (Table 3, Figure 5, and Supplementary Figure 5; 96 h), respectively. Then, the analysis of the mass spectrum of pellucidin A revealed the level of incorporation. For instance, the mass spectrum of pellucidin A at natural abundance displayed the molecular ion at *m/z* 388, and the fragment ions at *m/z* 194.0 and 179.0 Da with naturally abundant ions with ^13^C being compatible with intensities in the range of 0.07% (Figure 5 and Supplementary Figure 35). The feeding with L-(2-^13^C)-phenylalanine led to an increase in the percentage of ions at *m/z* 389 [M+1]^+.^, 195, 180, and 152 assigned to ^13^C-enriched pellucidin A with incorporation of one unit of L-(2-^13^C)-phenylalanine while the increasing percentage of the masses at 390 [M+2]^+.^ was attributed to pellucidin A enriched with two molecules of L-(2-^13^C)-phenylalanine (Figure 5). The level of incorporation of L-(2-^13^C)-phenylalanine in the time course experiments after 96 h of incubation led to a maximum of 0.72% enrichment in pellucidin A (Table 3).

#### Incorporation of (8-^13^C)-Cinnamic Acid and (8-^13^C)-Ferulic Acid

The GC-MS analysis of crude extracts of *P. pellucida* leaves incubated with (8-^13^C)-cinnamic acid and (8-^13^C)-ferulic acid showed the incorporation of these precursors into 2,4,5-trimethoxystyrene and pellucidin A. First, the incorporation of (8-^13^C)-cinnamic acid over the time course of 12–120 h resulted in the incorporation into 2,4,5-trimethoxystyrene and pellucidin A of a maximum of 2.89 and 1.32%, respectively (Table 3 and Supplementary Figure 5; 96 h). Then, the incubation of *P. pellucida* with (8-^13^C)-ferulic acid for 48 h led to its incorporation into both 2,4,5-trimethoxystyrene (0.69%) and pellucidin A (0.51%) (Table 3 and Supplementary Figure 6). The percentages of incorporation are in the range of incorporation of L-(2-^13^C)-phenylalanine into pellucidin A (Supplementary Figure 4) and are meaningful considering the number of steps required for L-phenylalanine to be initially converted to ferulic acid, and the additional further steps required to produce pellucidin A. Nevertheless, plants incubated for a period of time longer than 48 h showed signs of browning as compared with other feeding experiments and precluded further label chasing. The phenolic nature and the low solubility of (8-^13^C)-ferulic acid in aqueous solution may have contributed to difficulties in transportation to the biosynthetic site(s), as previously demonstrated (Davin et al., 2003; Vassao et al., 2006). In fact, several biosynthetic studies have shown low levels of cinnamic acid and ferulic acid incorporation, such as in the case of lignan podophyllotoxin in young plants (2–3 years) of *Podophyllum hexandrum*, with uptake of 0.04 and 0.053%, respectively (Jackson and Dewick, 1984). The incorporation of cinnamic acids in the (+)-lioniresinol found in *Lyonia ovalifolia* was in the range of 0.013–2.75% (Rahman et al., 2007). In the case of lignans such as (−)-*cis*-blechnic, (−)-*trans*-blechnic acids, and (−)-brainic acids occurring in the fern *Blechnum spicant*, the percentages of incorporation were significantly higher between 1.0 and 4.2% (Davin et al., 2003). The incorporation of the cinnamic acids is also described in studies of norlignan biosynthesis of (*Z*)-hinokiresinol in *Asparagus officinalis* (Suzuki et al., 2001) and agatharesinol from *Cryptomeria japonica* and *Agathis australis* (Imai et al., 2006). The individual administration of ^13^C-enriched cinnamic acid demonstrates that the side chain ^13^C-7, ^13^C-8, and ^13^C-9 atoms of cinnamic acid are incorporated into C-1 and C-3, C-2 and C-4, and C-5 of (*Z*)-hinokiresinol with variable percentages between 0.56 and 32.3%.

#### Incorporation of (8-^13^C)-2,4,5-Trimethoxycinnamic Acid

The GC-MS analyses of the crude extracts from *P. pellucida* leaves fed with (8-^13^C)-2,4,5-trimethoxycinnamic acid showed its sequential incorporation into 2,4,5-trimethoxystyrene and pellucidin A, reaching a maximum of 9.0 and 7.5%, respectively (Table 3 and Supplementary Figure 7; 96 h). The comparison between the relative abundances of the molecular ion of pellucidin A (*m/z* 388 Da, 0.34%; *m/z* 389 Da, [M+1]^+.^, 0.07%; *m/z* 390 Da, [M+2]^+.^, 0.03%) and the enriched version, revealed a higher relative intensity for [M+1]^+.^ (*m/z* 389 Da, 0.27%) and [M+2]^+.^ (*m/z* 390 Da, 0.04%) (Table 2 and Figures 4, 5).

Having characterized the *in vivo* incorporation of (8-^13^C)-2,4,5-trimethoxycinnamic acid to pellucidin A, experiments using enzymatic conversion were further conducted to evaluate the conversion of a series of unlabeled 2,4,5-trimethoxystyrene and 2,4,5-trihydroxystyrenes. The assays with enzymatic fractions from leaves of *P. pellucida* were carried out at varying temperatures, pH values and reaction times (data not shown). The extracts resulting from the assays were analyzed by HPLC to characterize the conversion of precursors to pellucidin A or related compounds. In fact, the comparison of the HPLC analyses of the control (enzyme extract) (Supplementary Figure 8) with the enzymatic fractions and the respective standards characterized the conversion of 2,4,5-trimethoxycinnamic acid to 2,4,5-trimethoxystyrene as the main product of the enzymatic conversion (Figure 4).

Further enzymatic conversion experiments were conducted to determine the level of specificity for the decarboxylation reaction. Thus, various cinnamic acids were tested to evaluate the formation of the respective styrenes including 2,4,5-trimethoxycinnamic acid, 3,4,5-trimethoxycinnamic acid, and 3,4-dimethoxycinnamic acid. Such reactions were observed in the first 30 min, after which the formation of the products remained practically constant up to 120 min without a significant increase in the yield (data not shown). Therefore, the decarboxylation of cinnamic acids in *P. pellucida* has apparently no specificity associated to the substitution pattern of the aromatic ring of cinnamic acids, although no compounds having electron withdrawing substituents at conjugated positions were evaluated. Enzymes capable of carrying out decarboxylation reactions have been described, as in the case of the ferulic acid decarboxylase from *Bacillus pumilus* and *Enterobacter* sp. (Gu et al., 2011), as well as *p*-coumaric acid decarboxylase purified from *Lactobacillus plantarum* (Cavin et al., 1997). The presence of these enzymes in plants is reported for species of *Catharanthus roseus, Nicotiana tabacum*, and *Daucus carota* (Takemoto and Achiwa, 1999). The action of decarboxylases in the biosynthesis of norlignans (*E*)- and (*Z*)-hinokiresinol in *A. officinalis* was also observed, in which the 4-coumaroyl 4-coumarate underwent a decarboxylation step followed by the formation of a bond between the carbons C7–C8′ leading to hinokiresinols (Suzuki et al., 2002; Suzuki and Umezawa, 2007).

#### Incorporation of (8-^13^C)-2,4,5-Trimethoxystyrene

The GC-MS analyses of the crude extracts of *P. pellucida* leaves resulting from incubation with (8-^13^C)-2,4,5-trimethoxystyrene showed its incorporation into pellucidin A with an incorporation of 12.8% at 96 h (Figure 5, Table 3, and Supplementary Figure 9). The incorporation of two molecules is noticeable with the relative intensity of the [M+2]^+.^ of labeled pellucidin A being near 0.17% of relative abundance, contrasting with 0.05 and 0.08% to the molecular ion and to [M+1]^+.^ ion of the natural version, respectively. In fact, the overall analysis of double-labeled pellucidin A, indicate the dilution effect of using L-phenylalanine, which is a primary precursor for coniferyl alcohol required for the biosynthesis of lignin, while for feeding labeled downstream intermediates in the formation of pellucidin A, the level of incorporations increases sharply. Thus, the formation of pellucidin A is confirmed to have 2,4,5-trimethoxystyrene as a direct precursor, and its formation should occur through a dimerization reaction between two 2,4,5-trimethoxystyrene units (Figure 4). This result contrasts with the biosynthetic suggestion for pellucidin A that precludes the possibility of dimerization between two 2,4,5-trimethoxystyrene units due to steric hindrance caused by the methoxy groups at positions 2/2′ of the aromatic rings (Bayma et al., 2000). However, as discussed above, pellucidin A has a *trans* configuration between the aromatic ring in the cyclobutane ring, which would minimize the repulsion between the methoxy groups at the 2/2′ positions.

#### Substrate Specificity for Pellucidin A Formation

According to the biosynthetic hypothesis, the incorporation of (8-^13^C)-2,4,5-trihydroxystyrene (Supplementary Figure 10; S10) would lead to the formation of a cyclobutane dimer (*m/z* 304 Da), followed by a series of methylations to produce pellucidin A (Bayma et al., 2000). The analysis of the GC-MS of the extracts resulting from the incubations did not detect the putative dimer produced from dimerization of (8-^13^C)-2,4,5-trihydroxystyrene. Feeding with the alternative labeled substrates (8-^13^C)-2,4,5-trihydroxyethylbenzene (S17), (8-^13^C)-3,4-dihydroxystyrene (S11) and (8-^13^C)-4-hydroxystyrene (S12) to determine whether methylations in the phenolic hydroxyl groups would take place as intermediate steps leading to pellucidin A (Figure 4) was also, not supported by GC-MS analysis of the crude extracts precluding such possibilities.

In addition to the ^13^C-cinnamic acids and ^13^C-styrenes used in the feeding experiments, unlabeled 3,4-dimethoxy- and 2,4,5-trimethoxypropenylbenzenes (Supplementary Figure 10) were also evaluated as substrates in enzymatic conversion using leaves of *P. pellucida*. The objective was to determine whether the enzymes from leaves of *P. pellucida* can produce pellucidin A analogs or related dimers, such as magnosalin or endiandrin A, dimers described from *Endiandra anthropophagorum* and *Piper cubeba*, respectively (Badheka et al., 1987; Davis et al., 2007). The analysis of extracts from *P. pellucida* resulting from incubation using enzymes with the different cinnamic acids and styrenes by GC-MS did not show the production of any other compound containing a cyclobutane ring.

#### Formation of Pellucidin A Under UV Light

Several examples of cyclobutane dimers of natural products including those resulting from pyrones and chalcone have been suggested as artifacts formed during compound isolation (Seidel et al., 2000; McCracken et al., 2012). Considering that this possibility could also be applied to the case of pellucidin A in *P. pellucida*, its formation during the extraction was investigated using the (8-^13^C)-2,4,5-trihydroxystyrene spiked in the solutions (see experimental). The analysis of relative intensities of the molecular ion peak, compared to the expected contribution of [M+1]^+.^ and [M+2]^+.^, did not support the formation of pellucidin A as an artifact. Nevertheless, the *in vivo* irradiation of plants under UV_365_ light led to an almost 200% increase in the content of pellucidin A (Supplementary Figure 2). Additional compounds that could be produced from dimerization of styrenes are pachypostaudin B, which has already been isolated from the extracts of *P. pellucida* (Kartika et al., 2020). However, pachypostaudins A and B are found as racemates in the extracts of *P. staudtii* bark, and were also obtained by thermal dimerization of 2,4,5-trimethoxystyrene (Ngadjui et al., 1989). The formation of pellucidin A could also be considered a photochemical artifact, but pachypostaudins were not detected in our study even in low amounts similar to pellucidin A in *P. pellucida*. Therefore, the biosynthesis of pellucidin A results from a more complex mechanism which requires studies of the molecular aspects, specificity for substrates, localization of biosynthetic steps and of its physiological role for the plant fitness.

Taken together these results indicate that the formation of pellucidin A in *P. pellucida* can be formed naturally having the L-phenylalanine followed by a series of steps of deamination forming cinnamic acid, then oxidation by phenolase, *O*-methylations leading to 2,4,5-trimethoxycinnamic acid, decarboxylation and then by a cycloaddition [2+2] reaction of 2,4,5-trimethoxystyrene to produce pellucidin A.

## Conclusion

The phytochemical study of *P. pellucida* led to the characterization of pellucidin A, 2,4,5-trimethoxystyrene, and dillapiol as previously described. Additionally, 2,4,5-trimethoxycinnamic acid, 2,4,5-trimethoxybenzaldehyde, sesamin and 5,6,7-trimethoxyflavone are to date undescribed compounds for *P. pellucida*. The chemical profiling of *P. pellucida* during ontogeny, revealed that 5,6,7-trimethoxyflavone is the major compound produced by seedlings of this species, while in the adult phase, a series of putative biosynthetic intermediates of pelludicin A such as cinnamic acid, styrenes, benzaldehydes, and pellucidin A itself, are produced. Furthermore, it was shown that damage to plantlets of *P. pellucida* by the herbivores *E. meditabunda*, *Monoplatus* sp. and an unknown Geometridae caterpillar did not affect in a detectable way the content of secondary compounds. Nevertheless, treatment with jasmonic acid was shown to induce the production of dillapiol and experiments with UV_365_ light led to an increase in the production of pellucidin A, by a photochemical [2+2] cycloaddition reaction of styrene.

Biosynthetic studies using ^13^C-labeled substrates with 2,4,5-trimethoxy substituents such as 2,4,5-trimethoxycinnamic acid and 2,4,5-trimethoxystyrene revealed that they were preferable over compounds having different substitution patterns in the aromatic ring to produce pellucidin A. The last stage of pellucidin A formation, *i.e.*, the dimerization of 2,4,5-trimethoxystyrene, is expected to take place by UV light, suggesting a potential photoprotective role for it similar to other flavones in plants.

## Data Availability Statement

The original contributions presented in the study are included in the article/Supplementary Material, further inquiries can be directed to the corresponding author/s.

## Author Contributions

MMM carried out the experimental work, interpreted the data, and contributed to writing the manuscript. MJK designed the research, interpreted the data, and wrote the manuscript. Both authors contributed to the article and approved the submitted version.

## Conflict of Interest

The authors declare that the research was conducted in the absence of any commercial or financial relationships that could be construed as a potential conflict of interest.
