# Supplementary material for: Biosynthesis of Pellucidin A in Peperomia pellucida (L.) HBK
Source: Front Plant Sci. 2021 Mar 22;12:641717. doi: 10.3389/fpls.2021.641717 (PMC8020151; doi:10.3389/fpls.2021.641717)
Supplement: Supplementary file 1 [file Data_Sheet_1.docx]

Supplementary Material

# Supplementary Data

### Spectrometric data and physical properties of isolated and synthetic compounds.

**5,6,7-Trimethoxyflavone (2).** Yellow solid; ^1^H NMR (300 MHz, CDCl_3_): δ 7.89 (m, 2H, H-2'/6'), 7.51(m, 3H, H-3'/4'/5'), 6.82 (s, 1H, H-8), 6.66 (s, 1H, H-3), 4.00 (s, 3H, 7-OCH_3_), 3.99 (s, 3H, 6-OCH_3_), 3.92 (s, 3H, 5-OCH_3_). ^13^C NMR (75 MHz, CDCl_3_): δ 177.1 (C-4), 161.0 (C-2), 157.7 (C-7), 154.5 (C-8a), 152.5 (C-5), 140.4 (C-6), 131.5 (C-1'), 131.2 (C-4'), 128.9 (C-5'/3'), 125.9 (C- 2'/6'), 112.9 (C-4a), 108.4 (C-3), 96.3 (C-8), 62.2 (5-OCH_3_), 61.5 (6-OCH_3_), 56.3 (7-OCH_3_).

**Pellucidin A (5):** White solid; ^1^H NMR (500 MHz, CDCl_3_): δ 6.98 (s, 2H, H-6/6'), 6.48 (s, 2H, H-3 /3'), 3.87 (m, 2H, H-7/7'), 3.86 (s, 6H, 5/5'-OCH_3_), 3.85 (s, 6H, 4/4'-OCH_3_), 3.75 (s, 6H, 2/2'-OCH_3_), 2.31 (dt, 5,1, 2,1 Hz, 2H, H-8α), 1.94 (dt, 5,1, 2,1 Hz, 2H, H-8β), ^13^C NMR (125 MHz, CDCl_3_): δ 151.0 (C-4/4'), 147.5 (C-2/2'), 143.1 (C-5/5'),124.6 (C-1/1'), 111.8 (C-6/6'), 97.8 (C-3/3'), 40.5 (C-7/7'), 56.6 (5/5'-OCH_3_), 56.5 (2/2'-OCH_3_), 56.2 (5/5'-OCH_3_), 27.0 (C-8/8').

**2,4,5-Trimethoxycinnamic acid (A1**): Yellow solid; ^1^H NMR (300 MHz, CDCl_3_): δ 8.11 (d, 15.7 Hz, 1H, H-7), 7.04 (s, 1H, H-6), 6.51 (s, 1H, H-3), 6.42 (d, 15.7 Hz, 1H, H-8), 3.94 (s, 3H, 2-OCH_3_), 3.89 (s, 3H, 4-OCH_3_), 3.88 (s, 3H, 5-OCH_3_). ^13^C NMR (75 MHz, CDCl_3_): δ 172.99 (C-9), 154.24 (C-2), 152.55 (C-4), 143.28 (C-5), 141.82 (C-1), 114.68 (C-8), 114.61 (C-3), 111.03 (C-7), 96.76 (C-6), 56.43 (2-OCH_3_), 56.32 (4-OCH_3_), 56.05 (5-OCH_3_). Yield: 96%.

**3-Hydroxy-4-methoxycinnamic acid** (Ferulic acid) (**A2**): White solid; ^1^H NMR (300 MHz, DMSO-d_6_): δ 7.75 (d, 15.1 Hz, 1H, H-7), 7.25 (bs, 1H, H-2), 7.09 (dd, 6.1 Hz, 1H, H-6), 6.82 (dd, 6.1 Hz, 1H, H-5), 6.40 (d, 15.1 Hz, 1H, H-8) 3.81 (s, 3H, 3-OCH_3_). ^13^C NMR (75 MHz, DMSO-d_6_): δ 168.50 (C-9), 149.42 (C-3), 148.27 (C-4), 144.96 (C-7), 126.20 (C-1), 123.11 (C-6), 115.97 (C-8), 111.43 (C-5), 111.43 (C-2). Yield: 94%.

**Cinnamic acid** (**A3**): White solid; ^1^H NMR (300 MHz, DMSO-d_6_): δ 7.64 (m, 2H, H-2 and H-6), 7.62 (d, 14.9 Hz, 1H, H-7), 7.39 (m, 3H, H-3, H-4 and H-5), 6.54 (d, 14.9 Hz, 1H, H-8). ^13^C NMR (75 MHz, DMSO-d_6_): δ 167.92 (C-9), 144.24 (C-1), 134.43 (C-7), 130.47 (C-4), 129.16 (C-2 and C-6), 128.39 (C-3 and C-5), 119.40 (C-8). Yield: 95%.

**3,4-Dimethoxycinnamic acid** (**A4**): White solid; ^1^H NMR (300 MHz, CDCl_3_): δ 7.77 (d, 15.9 Hz, 1H, H-7), 7.16 (dd, 5.5, 0.7 Hz, 1H, H-6), 7.08 (d, 0.7 Hz, 1H, H-2), 6.90 (d, 5.5 Hz, 1H, H-5), 6.35 (d, 15.9 Hz, 1H, H-8), 3.93 (s, 6H, 3-OCH_3_ and 4-OCH_3_). ^13^C NMR (75 MHz, CDCl_3_): δ 172.48 (C-9), 151.62 (C-4), 149.35 (C-3), 147.13 (C-7), 127.13 (C-1), 123.30 (C-6), 114.95 (C-5), 111.11 (C-8), 109.80 (C-2), 56.14 (4-OCH_3_), 56.04 (3-OCH_3_). Yield: 90%.

**2,4-Dimethoxycinnamic acid** (**A5**): White solid; ^1^H NMR (300 MHz, DMSO-d_6_): δ 7.77 (d, 14.4 Hz, 1H, H-7), 7.50 (d, 5.9 Hz, 1H, H-6), 6.51 (d, 5.9 Hz, 1H, H-5), 6.48 (s, 1H, H-3), 6.35 (d, 14.4 Hz, 1H, H-8), 3.79 (s, 3H, 2-OCH_3_), 3.74 (s, 3H, 4-OCH_3_). ^13C^ NMR (75 MHz, DMSO-d_6_): δ 168.50 (C-9), 162.71 (C-4), 159.46 (C-2), 139.01 (C-7), 130.00 (C-6), 116.41 (C-8), 115.87 (C-1), 106.13 (C-5), 98.41 (C-3), 55.74 (2-OCH_3_), 55.48 (4-OCH_3_). Yield: 95%.

**2,5-Dimethoxycinnamic acid** (**A6**): White solid; ^1^H NMR (300 MHz, DMSO-d_6_): δ 7.82 (d, 14.9 Hz, 1H, H-7), 7.16 (s, 1H, H-6), 6.93 (s, 1H, H-3 and H-4), 6.53 (d, 14.9 Hz, 1H, H-8), 3.76 (s, 3H, 2-OCH_3_), 3.70 (s, 3H, 5-OCH_3_). ^13^C NMR (75 MHz, DMSO-d_6_): δ 168.06 (C-9), 153.30 (C-5), 152.18 (C-2), 138.59 (C-7), 123.16 (C-1), 119.66 (C-8), 117.52 (C-4), 113.21 (C-3), 112.96 (C-6), 56.07 (2-OCH_3_), 55.57 (5-OCH_3_). Yield: 96%.

**2-Methoxycinnamic acid** (**A7**): White solid; ^1^H NMR (300 MHz, DMSO-d_6_): δ 7.87 (d, 14.8 Hz, 1H, H-7), 7.63 (t, 5.3 Hz, 1H, H-6), 7.38 (t, 5.9 Hz 1H, H-4), 7.03 (d, 5.9 Hz 1H, H-3), 6.97 (t, 5.3 Hz 1H, H-5), 6.52 (d, 14.8 Hz, 1H, H-8), 3.81 (s, 3H, 2-OCH_3_). ^13^C NMR (75 MHz, DMSO-d_6_): δ 168.09 (C-9), 157.85 (C-2), 138.87 (C-7), 131.86 (C-4), 128.52 (C-6), 122.21 (C-5), 120.82 (C-1), 119.36 (C-8), 111.74 (C-3), 55.66 (2-OCH_3_). Yield: 94%.

**3-Methoxycinnamic acid** (**A8**): White solid; ^1^H NMR (300 MHz, DMSO-d_6_): δ 7.59 (d, 15.3 Hz 1H, H-7), 7.25 (d, 4.0 Hz 1H, H-5), 7.20 (s, 2H, H-2 and H-4), 6.95 (d, 74.0 Hz, 1H, H-6), 6.55 (d, 15.3 Hz 1H, H-8), 3.74 (s, 3H, 3-OCH_3_). ^13^C NMR (75 MHz, DMSO-d_6_): δ 167.85 (C-9), 159.79 (C-3), 144.09 (C-1), 135.86 (C-7), 130.05 (C-5), 120.87 (C-6), 119.91 (C-8), 116.31 (C-4), 113.14 (C-2), 55.29 (3-OCH_3_). Yield: 94%.

**4-Methoxycinnamic acid** (**A9**): White solid; ^1^H NMR (300 MHz, DMSO-d_6_): δ 7.63 (d, 6.3 Hz, 2H, H-2 and H-6), 7.56 (d, 16.4 Hz, 1H, H-7) 6.97 (d, 6.3 Hz, 2H, H-3 and H-5), 6,38 (d, 16.4 Hz, 1H, H-8), 3,78 (s, 3H, 4-OCH_3_). ^13^C NMR (75 MHz, DMSO-d_6_): δ 167.99 (C-9), 161.07 (C-4), 143.91 (C-7), 130.05 (C-2 and C-6), 126.64 (C-1), 116.64 (C-8), 114.45 (C-3 and C-5), 55.34 (4-OCH_3_). Yield: 95%.

**2,4,5-Trihydroxycinnamic acid** (**A10**): Brown solid; ^1^H NMR (300 MHz, MeOD): δ 7.93 (d, 14.9 Hz, 1H, H-7), 7.01 (s, 1H, H-6), 6.64 (s, 1H, H-3), 6.30 (d, 14.9 Hz, 1H, H-8). ^13^C NMR (75 MHz, MeOD): δ 171.48 (C-9), 154.56 (C-4), 152.38 (C-2), 141.58 (C-5), 141.30 (C-1), 116.32 (C-8), 116.20 (C-3), 114.66 (C-7), 98.14 (C-6). Yield: 90%.

**3,4-Dihydroxycinnamic acid** (Caffeic acid) (**A11**): White solid; ^1^H NMR (300 MHz, MeOD): δ 7.54 (d, 15.2 Hz, 1H, H-7), 7.02 (s, 1H, H-2), 6.94 (d, 5.1 Hz, 1H, H-6), 6.78 (d, 5.1 Hz, 1H, H-5), 6.23 (d, 15.2 Hz, 1H, H-8). ^13^C NMR (75 MHz, MeOD): δ 171.02 (C-9), 149.44 (C-4), 147.02 (C-3), 146.78 (C-7), 127.79 (C-1), 122.84 (C-6), 118.04 (C-8), 115.51 (C-5), 115.07 (C-2). Yield: 91%.

**4-Hydroxycinnamic acid** (**A12**): White solid; ^1^H NMR (300 MHz, MeOD): δ 9.19 (d, 16.4 Hz, 1H, H-7) 8.99 (d, 6.0 Hz, 2H, H-2 and H-6), 8.38 (d, 6.0 Hz, 2H, H-3 and H-5), 7.86 (d, 16.4 Hz, 1H, H-8). ^13^C NMR (75 MHz, MeOD): δ 171.10 (C-9), 160.95 (C-4), 146.70 (C-7), 131.05 (C-2 and C-6), 127.14 (C-1), 116.95 (C-3 and C-5), 115.44 (C-8). Yield: 92%.

**4-Hydroxy-3,5-dimethoxycinnamic acid** (Synapic acid) (**A13**): Brown solid; ^1^H NMR (300 MHz, DMSO-d_6_): δ 8.99 (d, 14.8 Hz, 1H, H-7), 8.42 (s, 2H, H-2 and H-6), 7.89 (d, 14.8 Hz, 1H, H-8), 5.25 (s, 9H, 3-OCH_3_ and 5-OCH_3_). ^13^C NMR (75 MHz, DMSO-d_6_): δ 168.91 (C-9), 148.63 (C-3 and C-5), 145.63 (C-4), 138.65 (C-7), 125.42 (C-1), 116.65 (C-8), 106.43 (C-2 and C-6), 56.50 (3-OCH_3_ and 5-OCH_3_). Yield: 95%.

**2,4,5-Trimethoxyphenylpropanoic acid** (**A14**): White solid; ^1^H NMR (300 MHz, CDCl_3_): δ 6.73 (s, 1H, H-6), 6.51 (s, 1H, H-3), 3.87 (s, 3H, 2-OCH_3_), 3.82 (s, 3H, 4-OCH_3_), 3.80 (s, 3H, 5-OCH_3_), 2.90 (t, 5.1 Hz, 2H, H-7), 2.65 (t, 5.1 Hz, 2H, H-8). ^13^C NMR (75 MHz, CDCl_3_): δ 179.56 (C-9), 151.63 (C-2), 148.22 (C-4), 142.73 (C-5), 119.95 (C-1), 114.46 (C-6), 97.65 (C-3), 56.71 (2-OCH_3_), 56.26 (4-OCH_3_), 56.14 (5-OCH_3_), 34.47 (C-8), 25.52 (C-7). Yield: 96%.

**3,4-Methylenedioxycinnamic acid** (**A15**): White solid; ^1^H NMR (300 MHz, DMSO-d_6_): δ 7.50 (d, 14.8 Hz, 1H, H-7), 7.25 (s, 1H, H-2), 7.10 (d, 5.7 Hz, 1H, H-6), 6.88 (d, 5.7Hz, 1H, H-5), 6.36 (d, 14.8 Hz, 1H, H-8), 6.02 (s, 2H, OCH_2_O-3.4). ^13^C NMR (75 MHz, DMSO-d_6_): δ 168.03 (C-9), 149.28 (C-4), 148.18 (C-3), 144.00 (C-7), 128.81 (C-1), 124.69 (C-6), 117.19 (C-8), 108.53 (C-5), 106.74 (C-2), 101.70 (OCH_2_O-3.4). Yield: 96%.

**3,4,5-Trimethoxycinnamic acid** (**A16**): White solid; ^1^H NMR (300 MHz, DMSO-d_6_): δ 7.74 (d, 14.8 Hz, 1H, H-7), 6.79 (s, 2H, H-2 and H-6), 6.40 (d, 14.8 Hz, 1H, H-8), 3.90 (s, 9H, 3-OCH_3_, 4-OCH_3_ and 5-OCH_3_). ^13^C NMR (75 MHz, DMSO-d_6_): δ 172.43 (C-9), 153.59 (C-3 and C-5), 147.20 (C-4), 140.64 (C-7), 129.62 (C-1), 116.59 (C-8), 105.66 (C-2 and C-6), 61.31 (3-OCH_3_ and 5-OCH_3_) 56.31 (4-OCH_3_). Yield: 95%.

**2,4,5-Trimethoxystyrene** (**S1**): White solid; ^1^H NMR (300 MHz, CDCl_3_): δ 7.04 (dd, 15.5, 9.8 Hz, 1H, H-7), 7.01 (s, 1H, H-6), 6.50 (s, 1H, H-3), 5.63 (dd, 15.5, 1.9 Hz, 1H, H-8_T_), 5.18 (dd, 9.8, 1.9 Hz, 1H, H-8_C_) 3.89 (s, 3H, 2-OCH_3_), 3.87 (s, 3H, 4-OCH_3_), 3.83 (s, 3H, 5-OCH_3_). ^13^C NMR (75 MHz, CDCl_3_): δ 151.56 (C-2), 149.80 (C-4), 143.51 (C-5), 131.10 (C-1), 118.76 (C-6), 112.21 (C-3), 109.71 (C-7), 97.93 (C-8), 56.87 (2-OCH_3_), 56.65 (4-OCH_3_), 56.23 (5-OCH_3_). Yield: 91%.

**3-Hydroxy-4-methoxystyrene** (**S2**): Yellow liquid; ^1^H NMR (300 MHz, CDCl_3_): δ 6.83 (d, 4.4, 1.3 Hz, 1H, H-6), 6.80 (s, 1H, H-2), 6.78 (d, 4.4 Hz. 1H, H-5), 6.58 (dd, 15.3, 9.1 Hz, 1H, H-7), 5.51 (dd, 15.3, 0.7 Hz, 1H, H-8_T_), 5.04 (dd, 9.1, 0.7 Hz, 1H, H-8_C_) 3.76 (s, 3H, 3-OCH_3_). ^13^C NMR (75 MHz, CDCl_3_): δ 146.69 (C-4), 145.69 (C-3), 136.69 (C-7), 130.33 (C-1), 120.09 (C-6), 114.50 (C-5), 111.47 (C-8), 108.18 (C-2), 55.90 (3-OCH_3_). Yield: 94%.

**Styrene** (**S3**):White liquid; ^1^H NMR (300 MHz, CDCl_3_): δ 7.40 (m, 2H, H-2 and H-6), 7.33 (m, 2H, H-3 and H-5), 7.25 (m, 1H, H-4), 6.75 (dd, 14.3, 8.2 Hz, 1H, H-7), 5.76 (dd, 14.3, 1.1 Hz, 1H, H-8_T_), 5.24 (dd, 8.2, 1.1 Hz, 1H, H-8_C_). ^13^C NMR (75 MHz, CDCl_3_): δ 137.63 (C-1), 136.95 (C-7), 128.57 (C-3 and C-5), 126.28 (C-2 and C-6), 127.85 (C-4), 113.8 (C-8). Yield: 80%.

**3,4-Dimethoxystyrene** (**S4**): White solid; ^1^H NMR (300 MHz, CDCl_3_): δ 6.97 (dd, 4.5, 1.3 Hz, 1H, H-6), 6.93 (d, 1.3 Hz, 1H, H-2), 6.83 (d, 4.5Hz, 1H, H-5), 6.64 (dd, 15.5, 8.1 Hz, 1H, H-7), 5.64 (dd, 15.5, 0.9 Hz, 1H, H-8), 5.17 (dd, 8.1, 0.9 Hz, 1H, H-8C) 3.90 (s, 3H, 4-OCH_3_), 3.88 (s, 3H, 3-OCH_3_). ^13^C NMR (75 MHz, CDCl_3_): δ 149.12 (C-3 and C-4), 136.62 (C-7), 130.87 (C-1), 119.59 (C-6), 111.95 (C-5), 111.18 (C-8), 108.68 (C-2), 56.07 (4-OCH_3_), 55.96 (3-OCH_3_). Yield: 87%.

**2,4-Dimethoxystyrene** (**S5**): White solid; ^1^H NMR (300 MHz, CDCl_3_): δ 7.39 (d, 7.5 Hz, 1H, H-6), 6.99 (dd, 14.2, 9.4 Hz, 1H, H-7), 6.48 (dd, 7.5, 2.1 Hz, 1H, H-5), 6.43 (d, 2.1 Hz, 1H, H-3), 5.64 (dd, 14.2, 1.1 Hz, 1H, H-8), 5.15 (dd, 9.4, 1.1 Hz, 1H, H-8) 3.80 (s, 3H, 2-OCH_3_), 3.79 (s, 3H, 4-OCH_3_). ^13^C NMR (75 MHz, CDCl_3_): δ 160.56 (C-2), 157.83 (C-4), 131.25 (C-7), 127.24 (C-6), 119.86 (C-1), 112.22 (C-8), 104.72 (C-5), 98.36 (C-3), 55.33 (2-OCH_3_), 55.42 (4-OCH_3_). Yield: 87%.

**2,5-Dimethoxystyrene** (**S6**): White solid; ^1^H NMR (300 MHz, CDCl_3_): δ 7.25 (dd, 12.5, 7.3 Hz, 1H, H-7), 7.03 (d, 0.9 Hz, 1H, H-6), 6.79 (dd, 5.5, 0.9 Hz 1H, H-4), 6.78 (d, 5.5 Hz 1H, H-3), 5.75 (d, 12.5 Hz, 1H, H-8), 5.29 (d, 7.3 Hz, 1H, H-8) 3.82 (s, 3H, 2-OCH_3_), 3.78 (s, 3H, 5-OCH_3_). ^13^C NMR (75 MHz, CDCl_3_): δ 153.67 (C-5), 151.19 (C-2), 131.48 (C-7), 127.58 (C-1), 114.66 (C-3), 113.77 (C-4), 112.24 (C-8), 111.85 (C-6), 57.72 (5-OCH_3_), 56.23 (2-OCH_3_). Yield: 80%.

**2-Methoxystyrene** (**S7**): White solid; ^1^H NMR (300 MHz, CDCl_3_): δ 7.48 (dd, 7.4, 2.3 Hz, 1H, H-6), 7.21 (td, 7.4, 1.4 Hz, 1H, H-4), 7.06 (dd, 15.5, 6.3 Hz, 1H, H-7), 6.93 (td, 7.4, 1.2 Hz, 1H, H-5), 6.88 (dd, 7.4, 1.2 Hz, 1H, H-3), 5.76 (dd, 15.5, 1.3 Hz, 1H, H-8), 5.28 (dd, 6.3, 1.3 Hz, 1H, H-8) 3.84 (s, 3H, 2-OCH_3_). ^13^C NMR (75 MHz, CDCl_3_): δ 156.85 (C-2), 131.80 (C-4), 128.96 (C-7), 126.65 (C-6), 120.74 (C-5), 114.58 (C-1), 114.58 (C-8), 110.97 (C-3), 55.59 (2-OCH_3_). Yield: 89%.

**3-Methoxystyrene** (**S8**): White liquid; ^1^H NMR (300 MHz, CDCl_3_): δ 7.21 (td, 7.2, 0.8 Hz, 1H, H-5), 7.00 (dd, 7.2, 0.5 Hz, 1H, H-6), 6.97 (dd, 0.7, 0.5 Hz, 1H, H-2), 6.93 (dd, 7.2, 0.8 Hz, 1H, H-4), 6.80 (dd, 15.7, 9.9 Hz, 1H, H-7), 5.75 (dd, 15.7, 0.7 Hz, 1H, H-8), 5.24 (dd, 9.9, 0.7 Hz, 1H, H-8) 3.83 (s, 3H, 2-OCH_3_). ^13^C NMR (75 MHz, CDCl_3_): δ 159.83 (C-3), 136.80 (C-7), 139.05 (C-1), 129.51(C-5), 118.92 (C-6), 114.13 (C-8), 113.45 (C-4), 111.55 (C-2), 55.21 (3-OCH_3_). Yield: 90%.

**4-Methoxystyrene** (**S9**): White liquid; ^1^H NMR (300 MHz, CDCl_3_): δ 7.36 (d, 6.1 Hz, 2H, H-2 and H-6), 6.87 (d, 6.1 Hz, 2H, H-3 and H-5), 6.71 (dd, 12.0, 7.2 Hz, 1H, H-7), 5.63 (dd, 12.0, 1.4 Hz, 1H, H-8_T_), 5.14 (dd, 7.2, 1.4 Hz, 1H, H-8_C_), 3.80 (s, 3H, 4-OCH_3_). ^13^C NMR (75 MHz, CDCl_3_): δ 159.45 (C-4), 136.31 (C-7), 130.52 (C-1), 127.49 (C-2 and C-6), 114.00 (C-3 and C-5), 111.68 (C-8), 55.39 (4-OCH_3_). Yield: 93%.

**2,4,5-Trihydroxystyrene** (**S10**): Brown liquid; ^1^H NMR (300 MHz, MeOH): δ 8.99 (dd, 12.1, 6.1 Hz, 1H, H-7), 8.57(s, 1H, H-6), 8.14 (s, 1H, H-3), 7.32 (dd, 12.1, 2.2 Hz, 1H, H-8), 7.24 (dd, 6.1, 2.2 Hz, 1H, H-8). ^13^C NMR (75 MHz, MeOH): δ 155.06 (C-2), 148.36 (C-4), 140.29 (C-5), 136.83 (C-7), 115.88 (C-1), 114.57 (C-8), 113.20 (C-6), 102.23 (C-3). Yield: 83%.

**3,4-Dihydroxystyrene** (**S11**): Yellow solid; ^1^H NMR (300 MHz, MeOH): δ 8.45(s, 1H, H-6), 8.26 (s, 2H, H-2 and H-5), 8.11 (dd, 16.0, 9.1 Hz, 1H, H-7), 7.05 (dd, 16.0, 1.3 Hz, 1H, H-8), 6.53 (dd, 9.1, 1.3 Hz, 1H, H-8). ^13^C NMR (75 MHz, MeOH): δ 146.13 (C-4), 145.99 (C-3), 137.78 (C-7), 131.30 (C-1), 119.65 (C-6), 116.12 (C-5), 113.49 (C-8), 110.86 (C-2). Yield: 83%.

**4-Hydroxystyrene** (**S12**): White liquid; ^1^H NMR (300 MHz, CDCl_3_): δ 8.83 (d, 6.8 Hz, 2H, H-2 and H-6), 8.31 (d, 6.8 Hz, 2H, H-3 and H-5), 8.28 (dd, 13.9, 8.3 Hz, 1H, H-7), 7.15 (dd, 13.9, 0.5 Hz, 1H, H-8), 6.60 (dd, 8.3, 0.5 Hz, 1H, H-8_C_). ^13^C NMR (75 MHz, CDCl_3_): δ 158.36 (C-4), 137.78 (C-7), 130.74 (C-1), 128.41 (C-2 and C-6), 116.22 (C-3 and C-5), 110.77 (C-8). Yield: 83%.

**4-Hydroxy-3,5-dimethoxystyrene** (**S13**): White liquid; ^1^H NMR (300 MHz, CDCl_3_): δ 6.65 (s, 2H, H-2 and H-6), 6.64 (dd, 11.3, 6.7 Hz, 1H, H-7), 5.62 (dd, 11.3, 0.8 Hz, 1H, H-8_T_), 5.16 (dd, 6.7, 0.8 Hz, 1H, H-8_C_), 3.89 (s, 6H, 3-OCH_3_ and 5-OCH_3_). ^13^C NMR (75 MHz, CDCl_3_): δ 147.07 (C-3 and C-5), 136.81 (C-4), 134.83 (C-7), 129.17 (C-1), 111.78 (C-8), 103.06 (C-2 and C-6), 56.24 (3-OCH_3_ and 5-OCH_3_). Yield: 83%.

**3,4-Methylenedioxystyrene** (**S15**): White solid; ^1^H NMR (300 MHz, CDCl_3_): δ 6.95 (d, 0.8 Hz, 1H, H-2), 6.84 (dd, 4.4, 0.8 Hz, 1H, H-6), 6.76 (d, 4.4Hz, 1H, H-5), 6.66 (dd, 15.1, 8.7 Hz, 1H, H-7), 5.93 (s, 2H, OCH_2_O-3.4), 5.59 (dd, 15.1, 0.5 Hz, 1H, H-8), 5.13 (dd, 8.7, 0.5 Hz, 1H, H-8). ^13^C NMR (75 MHz, CDCl_3_): δ 148.00 (C-4), 147.36 (C-3), 136.36 (C-7), 132.13 (C-1), 121.00 (C-6), 111.95 (C-8), 108.18 (C-5), 105.40 (C-2), 101.04 (OCH_2_O-3.4). Yield: 93%.

**4-Allyl-2-methoxyphenol** (Eugenol) (**Eu**): Yellow liquid; ^1^H NMR (300 MHz, CDCl_3_): 8.83 (s, 2H, H-2 and H-5), δ 6.86 (s, 1H, H-6), 6.35 (dq, 11.4, 0.7 Hz, 1H, H-7), 6.14 (dq, 11.4, 5.5 Hz, 1H, H-8), 3.89 (s, 3H, 3-OCH_3_), 1.89 (dd, 5.5, 0.7 Hz, 3H, H-9). ^13^C NMR (75 MHz, CDCl_3_): δ 146.54 (C-3), 144.73 (C-4), 130.72 (C-8), 130.63 (C-1), 123.40 (C-7), 119.29 (C-6), 114.34 (C-5), 107.87 (C-2), 55.82 (3-OCH_3_), 18.33 (C-9). Yield: 83%.

**(*E*)-1,2,4-trimethoxy-5-(prop-1-en-1-yl)benzene** (*Iso*-asarone) (**IA**): Yellow liquid; ^1^H NMR (300 MHz, CDCl_3_): δ 6.93 (s, 1H, H-6), 6.68 (dq, 11.9, 0.9 Hz, 1H, H-7), 6.47 (s, 1H, H-3), 6.14 (dq, 11.9, 6.0 Hz, 1H, H-8), 3.85 (s, 3H, 2-OCH_3_), 3.83 (s, 3H, 4-OCH_3_), 3.78 (s, 3H, 5-OCH_3_), 1.89 (dd, 6.0, 0.9 Hz, 3H, H-9). ^13^C NMR (75 MHz, CDCl_3_): δ 150.64 (C-2), 148.74 (C-4), 143.31 (C-5), 125.07 (C-1), 124.13 (C-6), 118.93 (C-3), 109.82 (C-7), 97.88 (C-8), 56.54 (2-OCH_3_), 56.42 (4-OCH_3_), 56.01 (5-OCH_3_), 18.74 (C-9). Yield: 90%.

**3,4,5-Trimethoxystyrene** (**S16**): White solid; ^1^H NMR (300 MHz, CDCl_3_): δ 6.69 (dd, 13.5, 9.5 Hz, 1H, H-7), 6.64 (s, 2H, H-2 and H-6), 5.69 (d, 13.5 Hz, 1H, H-8_T_), 5.24 (d, 9.5 Hz, 1H, H-8_C_) 3.89 (s, 3H, 4-OCH_3_), 3.85 (s, 6H, 3-OCH_3_ and 5-OCH_3_). ^13^C NMR (75 MHz, CDCl_3_): δ 152.99 (C-3 and C-5), 137.63 (C-4), 136.47 (C-7), 133.03 (C-1), 112.95 (C-8), 102.92 (C-2 and C-6), 60.59 (4-OCH_3_), 55.73 (3-OCH_3_ and 5-OCH_3_). Yield: 90%.

**2,4,5-Trihydroxyethylbenzene** (**S17**): Brown liquid; ^1^H NMR (300 MHz, MeOH): δ 6.32 (s, 1H, H-6), 6.12 (s, 1H, H-3), 2.30 (q, 5.4 Hz, 2H, H-7), 0.95 (t, 5.4 Hz, 3H, H-8). ^13^C NMR (75 MHz, MeOH): δ 148.53 (C-2), 144.23 (C-4), 138.78 (C-5), 121.21 (C-1), 115.63 (C-3 and C-6), 23.48 (C-7), 15.12 (C-8). Yield: 87%.

**2,4,5-Trimethoxyethylbenzene** (**S18**): White solid; ^1^H NMR (300 MHz, CDCl_3_): δ 6.72 (s, 1H, H-6), 6.52 (s, 1H, H-3), 3.87 (s, 3H, 2-OCH_3_), 3.84 (s, 3H, 4-OCH_3_), 3.80 (s, 3H, 5-OCH_3_), 2.61 (q, 4.8 Hz, 2H, H-7), 1.20 (t, 4.8 Hz, 3H, H-8). ^13^C NMR (75 MHz, CDCl_3_): δ 151.46 (C-2), 147.61 (C-4), 143.07 (C-5), 121.51 (C-1), 113.68 (C-6), 98.20 (C-6), 56.82 (2-OCH_3_), 56.60 (4-OCH_3_), 56.41 (5-OCH_3_), 22.90 (C-7), 14.82 (C-8). Yield: 90%.

**List of Figures**

Figure S1. *Peperomia pellucida* (L.) HBK*.* and two natural herbivores used in the damaging experiments.

**Figure S2**. HPLC profiles of the crude extracts of *P. pellucida* leaves under different stress conditions. 2,4,5-trimethoxycinnamic acid (**1**); 2,4,5-trimethoxystyrene (**2**); 2,4,5-trimethoxybenzaldehyde (**3**); dillapiol (**4**), pellucidin A (**5**), sesamin (**6**) and 5,6,7-trimethoxyflavone (**7**). The detection wavelength was set at 260 nm.

Figure S3. Incorporation of ʟ-[2-^13^C]-phenylalanine into 2,4,5-trimethoxycinnamic acid and 2,4,5-trimethoxystyrene in *P. pellucida*. Each bar corresponds to the % of incorporation (mean ± standard deviation, N=3) from independent experiments.

Figure S4. Incorporation of ʟ-[2-^13^C]-phenylalanine into pellucidin A in *P. pellucida*. Each bar corresponds to the % of incorporation (mean ± standard deviation, N=3) from independent experiments.

Figure S5. Incorporation of [8-^13^C]-cinnamic acid into 2,4,5-trimethoxystyrene and pellucidin A in *P. pellucida*. Each bar corresponds to the % of incorporation (mean ± standard deviation, N=3) from independent experiments.

Figure S6. Incorporation of [8-^13^C]-ferulic acid into 2,4,5-trimethoxystyrene and pellucidin A in *P. pellucida*. Each bar corresponds to the % of incorporation (mean ± standard deviation, N=3) from independent experiments. The oxidation of tissues after 48 h did not allow longer incubation times.

Figure S7. Incorporation of [8-^13^C]-2,4,5-trimethoxycinnamic acid into pellucidin A and 2,4,5-trimethoxystyrene in *P. pellucida*. Each bar corresponds to the % of incorporation (mean ± standard deviation, N=3) from independent experiments.

**Figure S8.** HPLC analysis of conversion of 2,4,5-trimethoxycinnamic acid (1) to 2,4,5-trimethoxystyrene (2) using enzymatic fraction from leaves of *P. pellucida*. Detection wavelength was set at 260 nm. The control refers to the incubation of enzymatic fractions without substrates.

Figure S9. Incorporation of [8-^13^C]-2,4,5-trimethoxystyrene into pellucidin A in *P. pellucida*. The bars represent the standard deviation obtained from three independent experiments.

**Figure S10.** Structures of cinnamic acids (A1-A16) and styrenes (S1-S18) derivatives evaluated as biosynthetic intermediates in the formation of pellucidin A in *P. pellucida*.

**Figure S11.** Mass spectrum (EI) of 2,4,5-trimethoxycinnamic acid (**1**).

**Figure S12.** ^1^H NMR spectrum (CDCl_3_, 300 MHz) of 2,4,5-trimethoxycinnamic acid (**1**).

**Figure S13.** ^13^C NMR spectrum (CDCl_3_, 75 MHz) of 2,4,5-trimethoxycinnamic acid (**1**).

Figure S14. Mass spectrum (EI) of 2,4,5-trimethoxystyrene (2).

**Figure S15.** ^1^H NMR spectrum (CDCl_3_, 300 MHz) of 2,4,5-trimethoxystyrene (**2**).

**Figure S16.** ^13^C NMR spectrum (CDCl_3_, 75 MHz) 2,4,5-trimethoxystyrene (**2**).

Figure S17. Mass spectrum (EI) of 2,4,5-trimethoxybenzaldehyde (3).

**Figure** **S18.** ^1^H NMR spectrum (CDCl_3_, 300 MHz) of 2,4,5-trimethoxybenzaldehyde (**3**).

**Figura S19.** ^13^C NMR spectrum (CDCl_3_, 75 MHz) of 2,4,5-trimethoxybenzaldehyde (**3**).

Figure S20. Mass spectrum (EI) of dillapiol (4).

**Figure S21.** ^1^H NMR spectrum (CDCl_3_, 300 MHz) of dillapiol (**4**).

Figure S22. High resolution mass spectrum (ESI-TOF) of pellucidin A (5).

Figure S23. (A) Mass spectrum (EI) of pellucidin A; (B) expansion between the 300 to 400 Da region of the mass spectrum of pellucidin A (5).

**Figure S24.** ^1^H NMR spectrum (CDCl_3_, 500 MHz) of pellucidin A (**5**).

**Figure S25.** ^13^C NMR spectrum (CDCl_3_, 125 MHz) of pellucidin A (**5**).

Figure S26. Mass spectrum (EI) of sesamin (6).

**Figure S27.** ^1^H NMR spectrum (CDCl_3_, 300 MHz) of sesamin (**6**).

**Figure S28.** ^13^C NMR spectrum (CDCl_3_, 75 MHz) of sesamin (**6**).

Figure S29. Mass spectrum of high resolution (ESI-TOF) of 5,6,7-trimethoxyflavone (7).

Figure S30. Mass spectrum (EI) of 5,6,7-trimethoxyflavone (7).

**Figure S31**. ^1^H NMR spectrum (CDCl_3_, 300 MHz) of 5,6,7-trimethoxyflavone (**7**).

**Figure S32.** ^13^C NMR spectrum (CDCl_3_, 75 MHz) of 5,6,7-trimethoxyflavone (**7).**
**Figure S33.** Mass spectrum (EI) of 2,4,5-trimethoxycinnamic acid with natural abundance (A); After incorporation of ʟ-[2-^13^C]-phenylalanine (B).
**Figure S34.** Mass spectrum (EI) of the naturally occurring 2,4,5-trimethoxystyrene (A); after incorporation of ʟ-[2-^13^C]-phenylalanine (B).
**Figure S35.** Mass spectrum (EI) of pellucidin A with natural abundance (A, A1); after incorporation of ʟ-[2-^13^C]-phenylalanine (B, B1).
**Figure** **S36.** Mass spectrum (EI) of the naturally occurring 2,4,5-trimethoxystyrene (A); after incorporation of [8-^13^C]-2,4,5-trimethoxycinnamic acid (B). Expansion in the region of molecular ion [M^+.^] of 2,4,5-trimethoxycinnamic acid (B1).

Figure S37. Mass spectrum (EI) of pellucidin A with natural abundance (A); after incorporation of [8-^13^C]-2,4,5-trimethoxycinnamic acid (B). Expansion in the region of molecular ions of natural abundance pellucidin (A1) and after incorporation of [8-^13^C]-2,4,5-trimethoxycinnamic acid (B1).

Figure S38. Mass spectrum (EI) of pellucidin A with natural abundance (A); after incorporation of [8-^13^C]-2,4,5-trimethoxystyrene (B). Expansion in the region of molecular ions of pellucidin A in natural abundance (A1) and after incorporation of [8-^13^C]-2,4,5-trimethoxystyrene (B1).

Figure S1. *Peperomia pellucida* (L.) HBK*.* and two natural herbivores used in the damaging experiments. (Photo by MJK)

**Figure S2**. HPLC profiles of the crude extracts of *P. pellucida* leaves under different stress conditions. 2,4,5-trimethoxycinnamic acid (**1**); 2,4,5-trimethoxystyrene (**2**); 2,4,5-trimethoxybenzaldehyde (**3**); dillapiol (**4**), pellucidin A (**5**), sesamin (**6**), and 5,6,7-trimethoxyflavone (**7**). The detection wavelength was set at 260 nm.

Figure S3. Incorporation of ʟ-[2-^13^C]-phenylalanine into 2,4,5-trimethoxycinnamic acid and 2,4,5-trimethoxystyrene in *P. pellucida*. Each bar corresponds to the % of incorporation (mean ± standard deviation, N=3) from independent experiments.


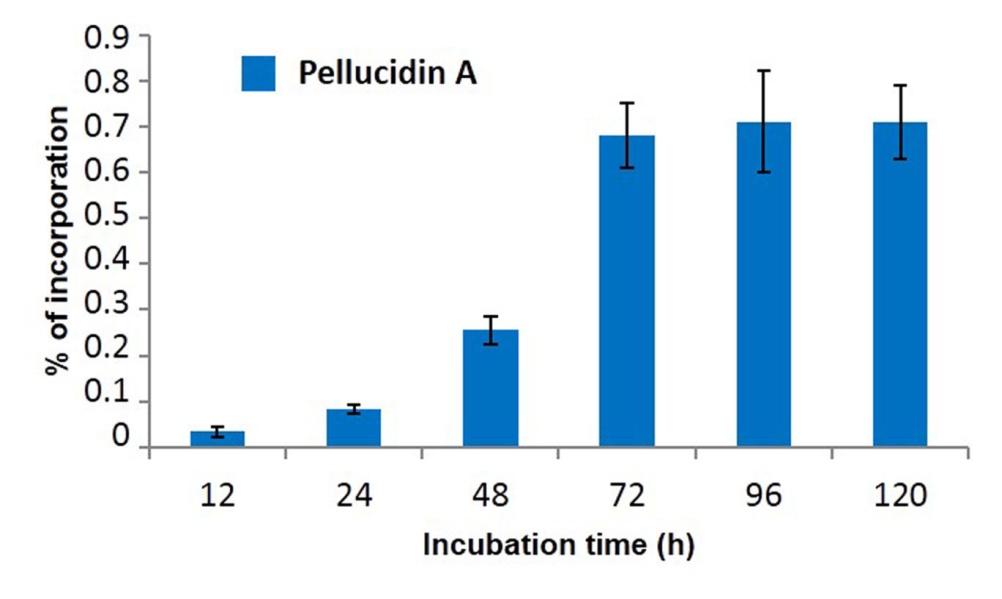


Figure S4. Incorporation of ʟ-[2-^13^C]-phenylalanine into pellucidin A in *P. pellucida*. Each bar corresponds to the % of incorporation (mean ± standard deviation, N=3) from independent experiments.

Figure S5. Incorporation of [8-^13^C]-cinnamic acid into 2,4,5-trimethoxystyrene and pellucidin A in *P. pellucida*. Each bar corresponds to the % of incorporation (mean ± standard deviation, N=3) from independent experiments.

Figure S6. Incorporation of [8-^13^C]-ferulic acid into 2,4,5-trimethoxystyrene and pellucidin A in *P. pellucida*. Each bar corresponds to the % of incorporation (mean ± standard deviation, N=3) from independent experiments. The oxidation of tissues after 48 h did not allow longer incubation times.

Figure S7. Incorporation of [8-^13^C]-2,4,5-trimethoxycinnamic acid into pellucidin A and 2,4,5-trimethoxystyrene in *P. pellucida*. Each bar corresponds to the % of incorporation (mean ± standard deviation, N=3) from independent experiments.

**Figure S8.** HPLC analysis of conversion of 2,4,5-trimethoxycinnamic acid (**1**) to 2,4,5-trimethoxystyrene (**2**) using enzymatic fraction from leaves of *P. pellucida*. Detection wavelength was set at 260 nm. The control refers to the incubation of enzymatic fractions without substrates.


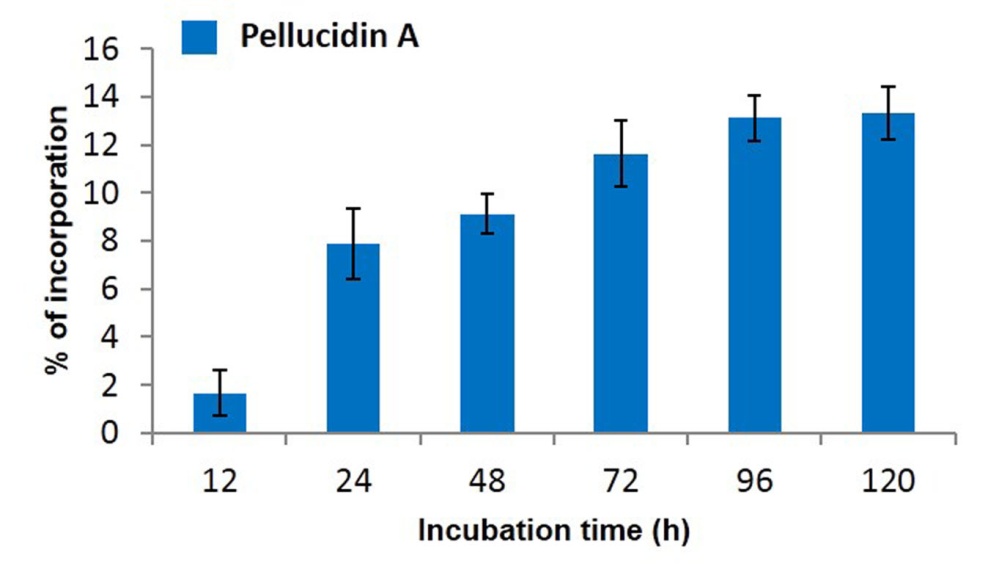


Figure S9. Incorporation of [8-^13^C]-2,4,5-trimethoxystyrene into pellucidin A in *P. pellucida*. Each bar corresponds to the % of incorporation (mean ± standard deviation, N=3) from independent experiments.

**Figure S10.** Structures of cinnamic acids (A1-A16) and styrenes (S1-S18) derivatives evaluated as biosynthetic intermediates in the formation of pellucidin A in *P. pellucida*.

Figure S11. Mass spectrum (EI) of 2,4,5-trimethoxycinnamic acid (1).

**Figure S12.** ^1^H NMR spectrum (CDCl_3_, 300 MHz) of 2,4,5-trimethoxycinnamic acid (**1**).

**Figure S13.** ^13^C NMR spectrum (CDCl_3_, 75 MHz) of 2,4,5-trimethoxycinnamic acid (**1**).

Figure S14. Mass spectrum (EI) of 2,4,5-trimethoxystyrene (2).

**Figure S15.** ^1^H NMR spectrum (CDCl_3_, 300 MHz) of 2,4,5-trimethoxystyrene (**2**).


**Figure S16.** ^13^C NMR spectrum (CDCl_3_, 75 MHz) of 2,4,5-trimethoxystyrene (**2**).
**Figure S17**. Mass spectrum (EI) of 2,4,5-trimethoxybenzaldehyde (**3**).

**Figure** **S18.** ^1^H NMR spectrum (CDCl_3_, 300 MHz) of 2,4,5-trimethoxybenzaldehyde (**3**).

**Figure S19.** ^13^C NMR spectrum (CDCl_3_, 75 MHz) of 2,4,5-trimethoxybenzaldehyde (**3**).

**Figure S20.** Mass spectrum (EI) of dillapiol (4).

**Figure S21.** ^1^H NMR spectrum (CDCl_3_, 300 MHz) of dillapiol (**4**).

Figure S22. High resolution mass spectrum (ESI-TOF) of pellucidin A (5).

Figure S23. (A) Mass spectrum (EI) of pellucidin A; (B) expansion between the 300 to 400 Da region of the mass spectrum of pellucidin A (5).

**Figure S24.** ^1^H NMR spectrum (CDCl_3_, 500 MHz) of pellucidin A (**5**).

**Figure S25.** ^13^C NMR spectrum (CDCl_3_, 125 MHz) of pellucidin A (**5**).

Figure S26. Mass spectrum (EI) of sesamin (6).

**Figure S27.** ^1^H NMR spectrum (CDCl_3_, 300 MHz) of sesamin (**6**).

**Figure S28.** ^13^C NMR spectrum (CDCl_3_, 75 MHz) of sesamin (**6**).

**Figure S29**. Mass spectrum of high resolution (ESI-TOF) of 5,6,7-trimethoxyflavone (**7**).

Figure S30. Mass spectrum (EI) of 5,6,7-trimethoxyflavone (7).

**Figure S31**. ^1^H NMR spectrum (CDCl_3_, 300 MHz) of 5,6,7-trimethoxyflavone (**7**).

**Figure S32.** ^13^C NMR spectrum (CDCl_3_, 75 MHz) of 5,6,7-trimethoxyflavone (**7**).


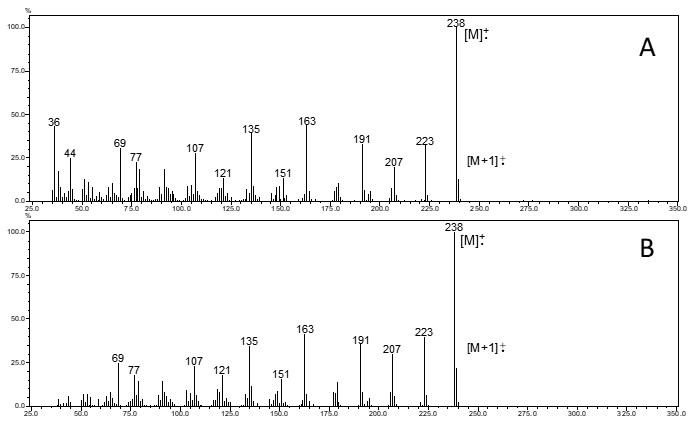

**Figure S33.** Mass spectrum (EI) of 2,4,5-trimethoxycinnamic acid with natural abundance (A); After incorporation of ʟ-[2-^13^C]-phenylalanine (B).


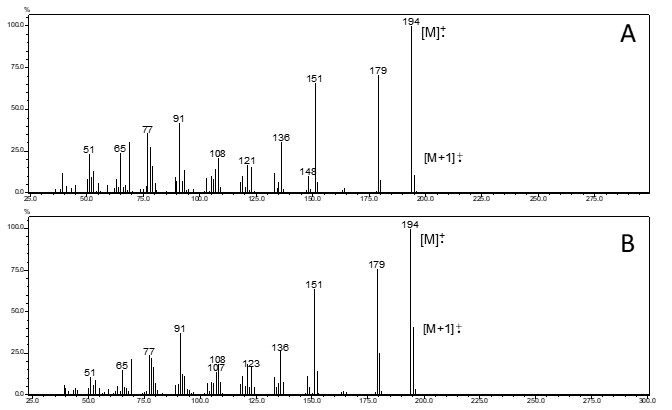


Figure S34. Mass spectrum (EI) of the naturally occurring 2,4,5-trimethoxystyrene (A); after incorporation of ʟ-[2-^13^C]-phenylalanine (B).

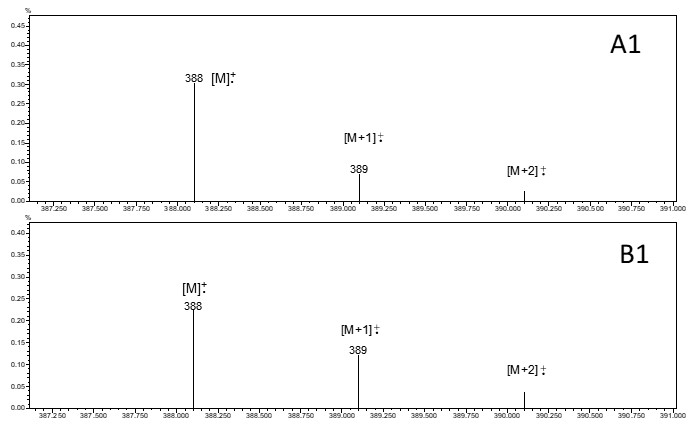


Figure S35. Mass spectrum (EI) of pellucidin A with natural abundance (A, A1); after incorporation of ʟ-[2-^13^C]-phenylalanine (B, B1).


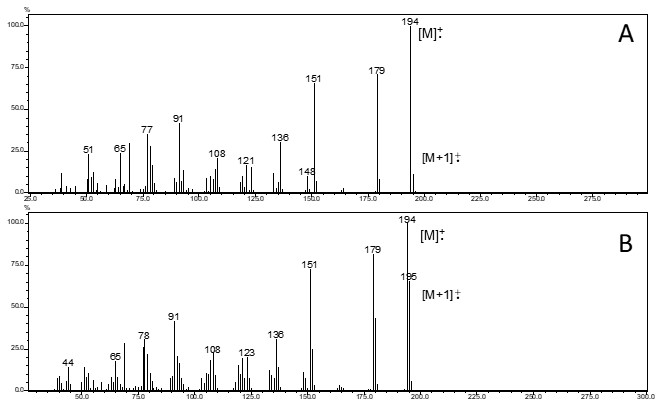

Figure S36. Mass spectrum (EI) of the naturally occurring 2,4,5-trimethoxystyrene (A); after incorporation of [8-^13^C]-2,4,5-trimethoxycinnamic acid (B). Expansion in the region of molecular ion [M^+.^] of 2,4,5-trimethoxycinnamic acid (B1).

**Figure** **S37**. Mass spectrum (EI) of pellucidin A with natural abundance (A) and after incorporation of [8-^13^C]-2,4,5-trimethoxycinnamic acid (B). Expansion in the region of molecular ions of natural abundance pellucidin (A1) and after incorporation of [8-^13^C]-2,4,5-trimethoxycinnamic acid (B1).

Figure S38. Mass spectrum (EI) of pellucidin A with natural abundance (A); after incorporation of [8-^13^C]-2,4,5-trimethoxystyrene (B). Expansion in the region of molecular ions of pellucidin A in natural abundance (A1) and after incorporation of [8-^13^C]-2,4,5-trimethoxystyrene (B1).
